# Defining functional variants associated with Alzheimer’s disease in the induced immune response

**DOI:** 10.1093/braincomms/fcab083

**Published:** 2021-04-19

**Authors:** Janet C Harwood, Ganna Leonenko, Rebecca Sims, Valentina Escott-Price, Julie Williams, Peter Holmans

**Affiliations:** 1 Division of Psychological Medicine and Clinical Neurosciences, School of Medicine, Cardiff University, Cardiff CF24 4HQ, UK; 2 UK Dementia Research Institute at Cardiff University, School of Medicine, Cardiff University, Cardiff CF24 4HQ, UK

**Keywords:** Alzheimer's disease, TWAS, monocytes, mitochondria, lipid metabolism

## Abstract

Defining the mechanisms involved in the aetiology of Alzheimer’s disease from genome-wide association studies alone is challenging since Alzheimer’s disease is polygenic and most genetic variants are non-coding. Non-coding Alzheimer’s disease risk variants can influence gene expression by affecting miRNA binding and those located within enhancers and within CTCF sites may influence gene expression through alterations in chromatin states. In addition, their function can be cell-type specific. They can function specifically in microglial enhancers thus affecting gene expression in the brain. Hence, transcriptome-wide association studies have been applied to test the genetic association between disease risk and cell-/tissue-specific gene expression. Many Alzheimer’s disease-associated loci are involved in the pathways of the innate immune system. Both microglia, the primary immune cells of the brain, and monocytes which can infiltrate the brain and differentiate into activated macrophages, have roles in neuroinflammation and β‐amyloid clearance through phagocytosis. In monocytes the function of regulatory variants can be context-specific after immune stimulation. To dissect the variants associated with Alzheimer’s disease in the context of monocytes, we utilized data from naïve monocytes and following immune stimulation *in vitro*, in combination with genome-wide association studies of Alzheimer’s disease in transcriptome-wide association studies. Of the nine genes with statistically independent transcriptome-wide association signals, seven are located in known Alzheimer’s disease risk loci: *BIN1, PTK2B, SPI1, MS4A4A, MS4A6E, APOE* and *PVR.* The transcriptome-wide association signal for *MS4A6E, PTK2B* and *PVR* and the direction of effect replicated in an independent genome-wide association studies. Our analysis identified two novel candidate genes for Alzheimer’s disease risk, *LACTB2* and *PLIN2/ADRP*. *LACTB2* replicated in a transcriptome-wide association study using independent expression weights. *LACTB2* and *PLIN2/ADRP* are involved in mitochondrial function and lipid metabolism, respectively. Comparison of transcriptome-wide association study results from monocytes, whole blood and brain showed that the signal for *PTK2B* is specific to blood and *MS4A6E* is specific to LPS stimulated monocytes.

## Teaser

Harwood et al. report a transcriptome-wide association study of monocyte gene expression on two Alzheimer's disease genome-wide association studies. As immunity is important in Alzheimer’s disease, this provides a biological interpretation in immune cells, of genome-wide association studies and implicates two novel genes, *PLIN2* and *LACTB2*, in Alzheimer’s disease.

## Graphical Abstract

**Graphical Abstract:**
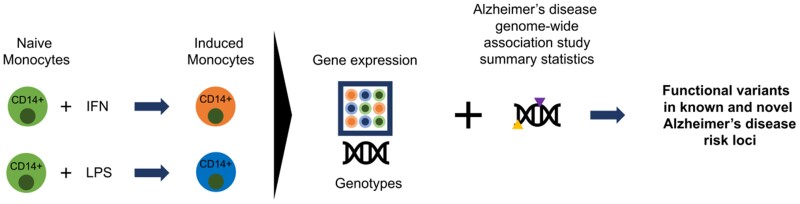

## Introduction

Alzheimer’s disease (AD) accounts for 60–70% of the dementias, affecting fifty million people worldwide. Alzheimer’s disease can occur sporadically in the population or may be inherited. Early-onset Alzheimer’s disease (EOAD), that accounts for ∼5% of Alzheimer’s disease cases, can be caused by autosomal dominant mutations that may be inherited or occur *de novo* in the *APP*,^1^ presenilin 1 (*PSEN1*)^2^ or presenilin 2 (*PSEN2*) genes.^3^ However, most cases of Alzheimer’s disease are late onset (LOAD) and occur after the age of 65 years. Although one of the strongest risk factors for Alzheimer’s disease (both EOAD and LOAD) is being a carrier of the E4 allele of the *APOE* gene,^4^ recent genome-wide association studies (GWAS) have shown that variants in more than 50 loci are implicated in LOAD,^5^ defining LOAD as a polygenic trait.^6^ Genes within Alzheimer’s disease risk loci are enriched in multiple diverse biological pathways: such as the immune response, cholesterol metabolism, amyloid protein processing and APP metabolism.^5^^,^^7^^,^^8^ However, the mechanisms involved in the mis-regulation of gene expression that leads to Alzheimer’s disease have not been elucidated. Many of the Alzheimer’s disease risk-associated variants are located in noncoding regions of the genome. These variants may be located in gene regulatory regions, affecting gene activity indirectly through regulation of their expression.^9^^,^^10^ For example, recent work has shown that Alzheimer’s disease risk variants can affect miRNA binding,^11^ can be located within enhancers and within CTCF sites, thus altering chromatin states^12^^,^^13^ and they can be located specifically in microglial enhancers^14^ thus affecting gene expression in the brain. Therefore, the next challenge is to draw together results of GWAS using functional analysis methods to understand the molecular basis of the associations. Methods have been developed to test the association between changes in cell/tissue-specific gene expression and disease by predicting functional/molecular phenotypes into GWAS data.^15–18^ Such analyses may be used to decipher the functional relevance of disease-associated loci, prioritising genes likely to be driving the association signal and to begin to define the mechanisms involved in their mis-regulation.

Microglia are the primary immune cells of the brain and have roles in innate immunity and in the development and maintenance of the central nervous system (CNS), maintaining neural circuits by synaptic pruning and by eliminating cellular debris. Microglia initiate an inflammatory response in the brain in response to amyloid-beta.^19^ However, as neuroinflammation inhibits neurogenesis^20^ it is thought that chronic activation of microglia may contribute to cognitive decline and neuropathogenesis in Alzheimer’s disease. In addition, recent studies have implicated the microglia-mediated innate immune response in the development of Alzheimer’s disease.^21^^,^^22^

As peripheral monocytes differentiate into microglia-like macrophages within the CNS, the purpose of this study was to refine the function of Alzheimer’s disease risk variants in the context of monocyte gene expression. This was based on the fact that there is an overlap between eQTLs in monocytes and Alzheimer’s disease risk variants.^23^ As many variants from Alzheimer’s disease-GWAS are in non-coding regions of the genome and the function of regulatory variants can be context - specific, they could be specific to immune-stimulated cells. We have used published expression and genetic data from monocytes at baseline and after induction with LPS and IFN^24^ with the largest available Alzheimer’s disease GWAS summary statistics^8^ to study genetically controlled gene expression in monocytes. Given that the study of the correct tissue is important in identifying disease-relevant associations,^25^ integrating monocyte expression with GWAS data using transcriptome-wide association studies (TWAS) was expected to give insights into the genes within the immune response pathways that contribute to Alzheimer’s disease risk, along with the direction of effect and insight into the mechanisms involved the aetiology of Alzheimer’s disease.

## Materials and methods

### Monocyte data

Monocyte expression data^24^ was downloaded from array express (URL.1). The corresponding genetic data were obtained from Dr Julian Knight University of Oxford under Licence.^24^^,^^26–28^ For replication, data from the Cardiogenics transcriptomic study (CTS) were downloaded from the European Genome-phenome Archive (URL.2).

### Fairfax monocyte expression data

Array Address Ids of the filtered probe set, previously quality controlled by Fairfax et al.^24^ were extracted (15421 probes) from the Fairfax et al.’s Supplementary file: 1246949stableS1.xlsx (URL.3). An in-house script was used to convert Array Address Ids to Illumina Probe ids using the Illumina HumanHT-12_V4_0_R1_15002873_B array annotation information (URL.4). Probes mapping to single genes for the 228 individuals with matched expression data available across the naïve (CD14) and induced monocytes (LPS2, LPS24, IFN) were extracted. Probes were mapped to GRCh37 (hg19) using the illumina_humanht_12_v4 chip annotation information using the Biomart package (URL.5) in R. 12, 469 expression probes were collapsed to 9743 genes using the ‘collapseRows’ function from the WGCA package (URL.6) using the MaxMean method^29^ in R (version 3.5.1).

### CTS monocyte expression data

We used published, quality controlled CTS monocyte expression data: File EGAF00000183279/monocyte_expression_subset.txt.^30^ We extracted 20 356 autosomal single gene probes and mapped them to the GRCh37 (hg19) genome assembly using the illumina_humanref_8_v3 annotation information using the Biomart package in R (URL.5), expression probes for were collapsed to 15 344 genes using the ‘collapseRows’ function from the WGCA package (URL.6) using the MaxMean method^29^ in R (version 3.5.1).

### Monocyte genetic data

Genetic data files from the Fairfax and the Cardiogenics (CTS) data sets were aligned to the GRCh37 (hg19) using the Liftover tool (URL.7). Plink 1.9 (URL.8) was used for standard Quality-Control analysis.^31^ Genetic data for the 228 individuals with matched expression data across all monocytes in the Fairfax data set (CD14, LPS2, LPS24 and IFN) was extracted. Genetic data for all 758 individuals from the CTS study was used.

In both the Fairfax and the CTS genetic data sets, SNPs were removed if their minor allele frequency (MAF) < 0.01, missingness of genotypes ≥ 0.02 or Hardy−Weinberg Equilibrium (HWE) <10^−6^. For the Fairfax study, a total of 625 793 variants were retained for 228 people. For the CTS study, a total of 509 870 SNPs were retained for 758 people.

### Genetic and environmental risk for Alzheimer’s disease genetic data

Genotypic data from the Genetic and Environmental Risk for Alzheimer’s disease (GERAD) consortium, without the National blood donor (NBS) cohort, imputed on the Haplotype Research Consortium panel,^32^ were used to test whether the TWAS association with PVR is independent of APOE genotype (see below). Tools from the McCarthy group (URL.9) and Plink 1.9 were used for quality control of GERAD data. SNPs were removed if their MAF < 0.01, missingness of genotypes ≥0.05 or HWE < 10^−6^, a total of 5 985 900 SNPs were retained for 10 687 people (3332 cases, 7355 controls).

### Logistic regression models for testing independence of PVR TWAS from APOE

To test whether the PVR prediction from TWAS is independent of APOE genotype, allelic scores for the PVR gene were derived using the PVR expression weight from LPS24 monocytes and genetic data from the GERAD study, using the make_score.R script from the FUSION software^16^ and Plink 1.9. APOE genotypes (coded according to the presence of 0, 1 or 2 APOE e4 alleles) were used as a covariate in a logistic regression in R (version 3.5.1) of Alzheimer’s disease status on genetically predicted PVR expression.

### Alzheimer’s disease summary statistics

International Genomics of Alzheimer’s Project (IGAP) is a large three-stage study based upon GWAS on individuals of European ancestry. In Stage 1, IGAP used genotyped and imputed data on 11 480 632 single nucleotide polymorphisms (SNPs) to meta-analyse GWAS datasets consisting of 21 982 Alzheimer’s disease cases and 41 944 cognitively normal controls from four consortia: the Alzheimer Disease Genetics Consortium (ADGC); The European Alzheimer’s disease Initiative (EADI); The Cohorts for Heart and Aging Research in Genomic Epidemiology Consortium (CHARGE) and The Genetic and Environmental Risk in Alzheimer’s disease Consortium Genetic and Environmental Risk in Alzheimer’s disease/Defining Genetic, Polygenic and Environmental Risk for Alzheimer’s Disease Consortium (GERAD/PERADES). In Stage 2, 11 632 SNPs were genotyped and tested for association in an independent set of 8362 Alzheimer’s disease cases and 10 483 controls. Meta-analysis of variants selected for analysis in Stage 3A (*n* = 11 666) or Stage 3B (*n* = 30 511) samples brought the final sample to 35 274 clinical and autopsy-documented Alzheimer’s disease cases and 59 163 controls.

In the work presented here, GWAS Stage 1 summary statistics from Kunkle et al.^8^ were used. Common variants from this large and powerful study (21 982 cases and 41 944 controls) were filtered using the munge_sumstats.py (v2.7.13) script from the LD Score Regression (LDSC) software (URL.10) and the hapmap 3.0 reference panel (URL.11 resulting in 1 207 073 SNPs). Common variants from UK Biobank GWAS on parental Alzheimer’s disease: maternal and paternal biobank data without IGAP (cases = 42 034, controls = 272 244)^33^ were filtered as described above, resulting in 1 188 072 SNPs.

### Expression weights

Expression weights for monocyte data were computed using the R script: FUSION.compute_weights.R from the FUSION software.^16^ For the Fairfax data, the binary plink files for the 228 individuals with matched expression data across all 4 monocytes were used. Similarly, the CTS data binary plink files for the 758 individuals and the corresponding expression data were used. Weights were packaged using the script OP_packaging_fusion_weights.R from Oliver Pain (URL.12).

GTEx7 and common mind consortium (CMC) expression weights and the reference panel from the 1000 Genomes European population were downloaded from the FUSION web site (URL.13).

UpSetR (URL.14) was used to determine the number of genes computed in the expression weights in common and unique to each of the stimulation conditions applied to Fairfax monocytes.

### Transcriptome-wide association studies

Expression weights were used in TWAS for autosomal chromosomes and excluding the MHC region, with the Alzheimer’s disease summary statistics using the R script FUSION.assoc_test.R from the FUSION software.^16^ Results were corrected for multiple testing of multiple genes within each tissue using the Bonferroni method. Where a significant TWAS association was obtained from multiple genes in a locus (±500 kb), conditional analysis was used to obtain statistically independent signals using the FUSION.postprocess.R script and default parameters.

### Gene-wide analysis of the Kunkle et al. summary statistics

We used the most recent version (v1.08b) of MAGMA, to obtain gene-wide *P*-values for LACTB2 and PLIN2 from the Kunkle et al. GWAS summary statistics. SNPs were assigned to genes if they lay within the gene boundaries (as defined by NCBI) and the MAGMA ‘mean’ method was used to derive the gene-wide association statistic (the sum of the squared *Z* statistics for individual SNPs).

### Statistical analyses

As described above, we used FUSION software^16^ for computing TWAS weights and for performing association tests, using the largest available GWAS and monocyte expression data. Bonferroni correction for the number of genes analysed in each tissue was used to determine significance of TWAS associations. Plink v1.9^34^ was used for SNP quality-control analysis, and MAGMA (v1.08)^35^ to obtain gene-wide *P*-values and Logistic regression was performed in R.

We used STREGA guidelines to report our study.^36^

### URLs

1. https://www.ebi.ac.uk/arrayexpress/experiments/E-MTAB-2232/. Accessed 22 April 2021
2. https://www.ebi.ac.uk/ega/studies/EGAS00001000411. Accessed 22 April 2021
3. Fairfax et al.’ supplementary material: https://science.sciencemag.org/content/suppl/2014/03/05/343.6175.1246949.DC1?_ga=2.250430726.142617004.1580743814-1392779675.1580743814. Accessed 22 April 2021
4. Illumina HumanHT-12_V4_0_R1_15002873_B array: https://www.ebi.ac.uk/arrayexpress/files/A-MEXP-2210/A-MEXP-2210.adf.txt. Accessed 22 April 2021
5. Biomart: https://bioconductor.org/packages/release/bioc/html/biomaRt.html. Accessed 22 April 2021
6. WGCNA: https://horvath.genetics.ucla.edu/html/CoexpressionNetwork/Rpackages/WGCNA/. Accessed 22 April 2021
7. Liftover tool: https://genome.ucsc.edu/cgi-bin/hgLiftOver. Accessed 22 April 2021
8. Plink 1.9: http://www.cog-genomics.org/plink/1.9/. Accessed 22 April 2021
9. McCarthy group tools: https://www.well.ox.ac.uk/∼wrayner/tools/#Checking. Accessed 22 April 2021
10. LD Score Regression (LDSC) software: V 1.0.0: https://github.com/bulik/ldsc. Accessed 22 April 2021
11. HapMap 3: https://www.sanger.ac.uk/resources/downloads/human/hapmap3.html. Accessed 22 April 2021
12. Oliver Pain: OP_packaging_fusion_weights.R: https://github.com/opain/Calculating-FUSION-TWAS-weights-pipeline. Accessed 22 April 2021
13. FUSION software: http://gusevlab.org/projects/fusion/. Accessed 22 April 2021
14. UpSetR: https://cran.r-project.org/web/packages/UpSetR. Accessed 22 April 2021
15. Common mind consortium: https://www.nimhgenetics.org/resources/commonmind. Accessed 22 April 2021

### Data availability

Monocyte TWAS weights are available at https://github.com/janetcharwood/MONOCYTE_TWAS URLs for publicly available datasets are given in the URL section.

## Results

### TWAS using Fairfax monocyte data

**Figure 1:**
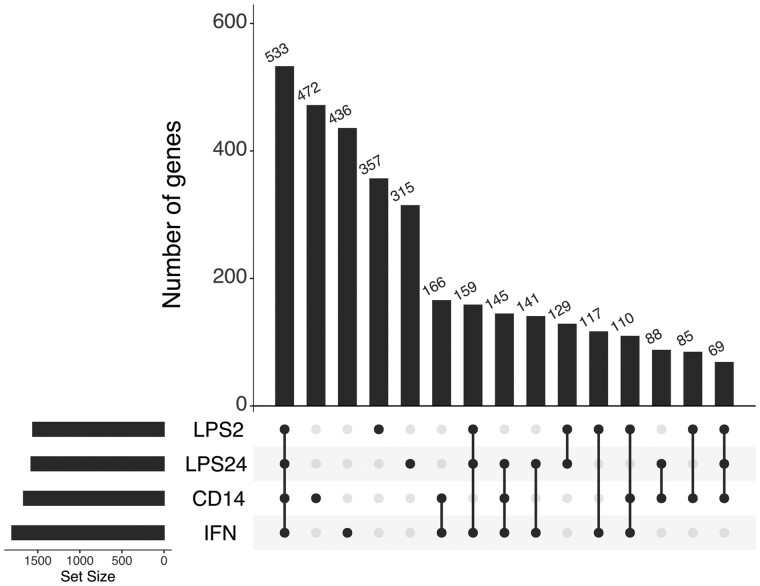
**Genes for which *cis*-heritable expression was computed overlap between naïve and induced monocyte cell types.** UpSet plots show the numbers of genes in common and unique to each of the monocytes: naïve CD14+ cells (CD14), CD14+ cells induced with LPS for 2 h (LPS2), CD14+ cells induced with LPS for 24 h (LPS24) and IFN-induced CD14+ cells (IFN).

Using the FUSION software,^16^ we computed expression weights for monocytes at baseline and after and immune-stimulation with LPS and IFN using published genetic and expression data from 228 healthy European individuals.^24^ Monocytes from the same individuals at baseline and after immune stimulation *in vitro* using LPS or interferon-γ (IFN) were tested; naïve monocytes expressing CD14 (CD14+), stimulated with lipopolysaccharide for 2 h (LPS2) and 24 h (LPS24) and with IFN. In each case, 1600–1800 genes were modelled with significant *cis*-heritable expression (Supplementary Table 1). The overlap in the number of genes for which *cis*-heritable expression was computed in each of the monocytes is shown in the UpSet plot (Fig. 1). Significant TWAS association signals were defined as those with *P* < 0.05 after Bonferroni correction for the number of genes for which TWAS was performed in each tissue/cell population.

**Figure 2:**
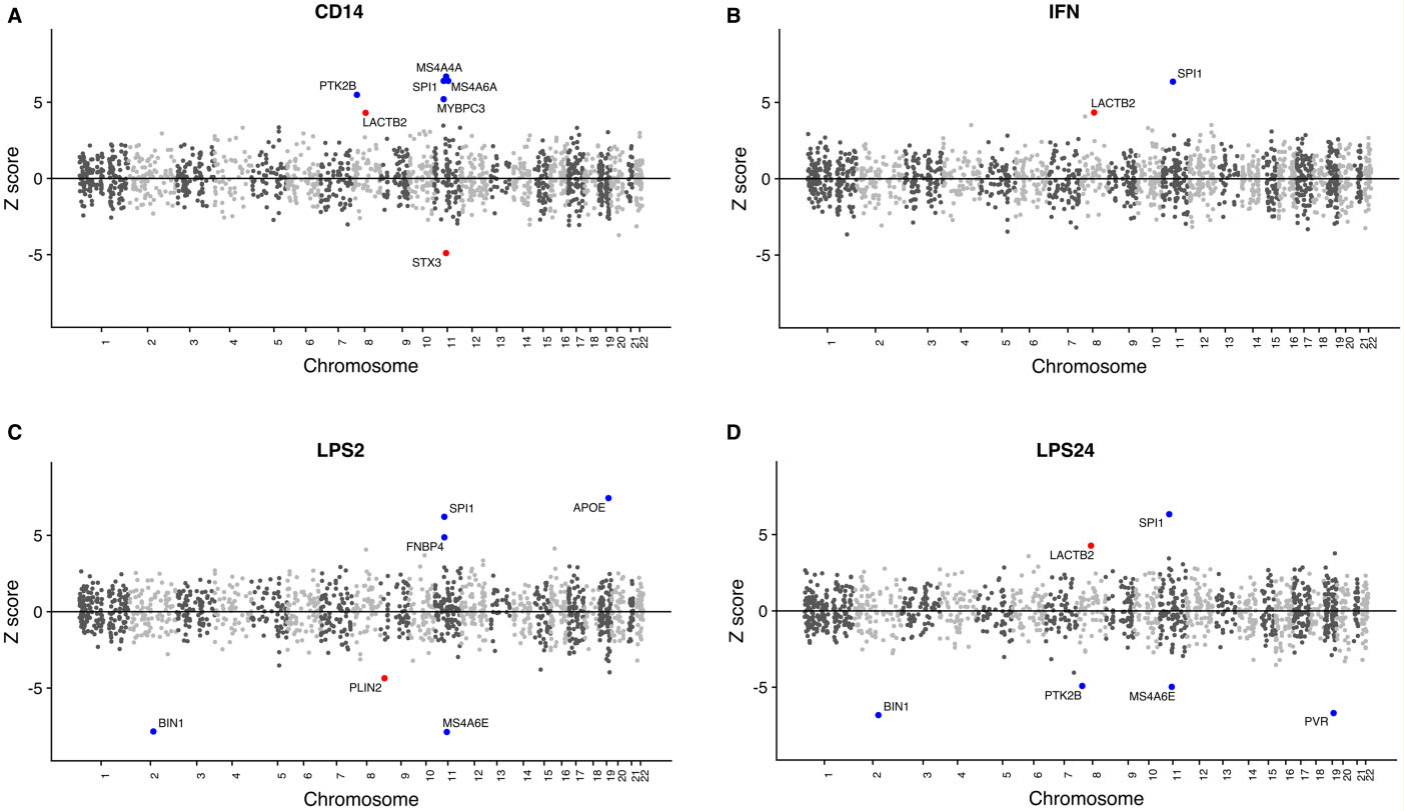
**Mirrored Manhattan plots of TWAS results. Genes are represented by coloured points plotted on the *x* axis by chromosome and genomic location.** The *y* axis is the *Z*-score of the association between gene expression and Alzheimer’s disease in naïve CD14+ cells (**A**), CD14+ cells induced with IFN (**B**), CD14+ cells induced with LPS for 2 h (**C**) and 24 h (**D**). Genes that show significant association after Bonferroni correction for multiple testing are shown with red (novel Alzheimer’s disease genes) and blue points (genes in a known Alzheimer’s disease locus) and named with black text.

Using the latest summary statistics for diagnosed Alzheimer’s disease^8^ and the monocyte expression weights described above, we ran TWAS analysis on naïve (CD14+) and following immune stimulation *in vitro* using LPS or IFN (Supplementary Table 2) (LPS2, LPS24 and IFN) using FUSION software.^16^ Across the monocytes tested, a significant TWAS association signal was generated for 13 genes (Fig. 2 and Table 1).

**Table 1:** Transcriptome-wide association study (TWAS) test statistics for thirteen significantly associated genes for Alzheimer's disease in monocytes

Monocyte	Gene	Chromosome	Gene start	Gene end	K_TWAS.Z	M_TWAS.Z	K_TWAS.P	M_TWAS.P
CD14+	***LACTB2***	8	71547553	71581409	4.305	0.861	1.67E−05	3.89E−01
CD14+	PTK2B**	8	27168999	27316903	5.485	4.325	4.14E−08	1.52E−05
CD14+	*MS4A4A*	11	60048014	60076445	6.683	3.200	2.33E−11	1.37E−03
CD14+	*MS4A6A*	11	59939081	59952139	6.431	2.431	1.27E−10	1.51E−02
CD14+	*MYBPC3*	11	47352957	47374253	5.204	1.093	1.95E−07	2.74E−01
CD14+	*SPI1*	11	47376411	47400127	6.397	1.279	1.58E−10	2.01E−01
CD14+	*STX3*	11	59480929	59573354	−4.898	−2.302	9.66E−07	2.13E−02
IFN	***LACTB2***	8	71547553	71581409	4.361	1.621	1.29E−05	1.05E−01
IFN	*SPI1*	11	47376411	47400127	6.337	1.207	2.35E−10	2.27E−01
IFN	APOE*	19	45409011	45412650	−2.495	−6.039	1.26E−02	1.55E−09
IFN	ZNF668*	16	31072813	31073451	1.759	4.355	7.86E−02	1.33E−05
LPS2	*BIN1*	2	127805603	127864931	−7.843	−3.403	4.40E−15	6.66E−04
LPS2	***PLIN2***	9	19108373	19149288	−4.356	−1.918	1.33E−05	5.51E−02
LPS2	MS4A6E**	11	60102304	60164069	−7.879	−4.314	3.31E−15	1.61E−05
LPS2	*SPI1*	11	47376411	47400127	6.218	1.327	5.04E−10	1.84E−01
LPS2	*FNBP4*	11	47738072	47788995	4.873	1.118	1.10E−06	2.63E−01
LPS2	*APOE*	19	45409011	45412650	7.441	4.099	1.00E−13	4.16E−05
LPS24	*BIN1*	2	127805603	127864931	−6.825	−2.615	8.78E−12	8.92E−03
LPS24	*PTK2B*	8	27168999	27316903	−4.916	−3.312	8.85E−07	9.28E−04
LPS24	***LACTB2***	8	71547553	71581409	4.274	2.262	1.92E−05	2.37E−02
LPS24	*SPI1*	11	47376411	47400127	6.333	1.219	2.41E−10	2.23E−01
LPS24	*MS4A6E*	11	60102304	60164069	−4.974	−2.402	6.57E−07	1.63E−02
LPS24	PVR**	19	45147098	45166850	−6.691	−5.495	2.21E−11	3.90E−08
LPS24	FOSB*	19	45971253	45978437	−1.571	−4.737	1.16E−01	2.17E−06

Genes showing TWAS significant associations (*P* < 0.05 after Bonferroni correction for the number of genes for which TWAS was performed in each tissue). TWAS.P values shown are not corrected for multiple testing. K_TWAS.Z and M_TWAS.Z denote the gene-level TWAS *Z*-score using Kunkle 2019^8^ and Marioni summary statistics,^33^ respectively. Genes without asterisks are TWAS significant using Kunkle summary statistics.

* TWAS significant genes using Marioni summary statistics.

** TWAS significant genes in both Kunkle and Marioni TWAS. TWAS significant genes that are novel candidate genes for Alzheimer’s disease are shown in bold.

Of the TWAS significant genes across the monocytes, those for which expression weights could be derived, and thus TWAS analyses performed, are shown in Supplementary Table 3. Three of these genes: *STX3*, *LACTB2* and *PLIN2* had novel significant associations with Alzheimer’s disease. The remaining 10 genes (*APOE*, *BIN1*, *FNBP4*, *MS4A4A*, *MS4A6E*, *MYBPC3*, *PTK2B*, *PVR*, *SPI1* and *STX3*) are located in published loci significantly associated with Alzheimer’s disease risk from GWAS, giving confidence that we are detecting effects of genuine disease relevance using monocyte TWAS. Where there were multiple-associated features at the same locus, we identified those that were conditionally independent using the post-processing script in the FUSION software. TWAS significant genes in the same region (those within a 100-kb window) were aggregated and conditional analysis on the other TWAS signals at the locus was undertaken for these regions (Supplementary Table 4 and Supplementary material—Conditional analysis plots). *STX3*, one of the novel TWAS significant genes was dropped after conditional analysis along with three of the genes in Alzheimer’s disease risk loci: *FNBP4*, *MYBPC3* and *MS4A6A*.

Hence, using monocyte expression weights derived from the Fairfax et al. data, we identified nine genes with statistically independent TWAS signals (conditional *P* ≤ 0.05). *LACTB2* and *PLIN2* had novel significant associations with Alzheimer’s disease and *BIN1*, *PTK2B*, *SPI1*, *MS4A4A*, *MS4A6E*, *APOE* and *PVR* are in known Alzheimer’s disease risk loci from GWAS.

### Comparison with TWAS from independent monocyte data

We replicated the TWAS using an independent monocyte expression data set from the CTS.^30^ In this study, CD14+ monocytes were purified from 758 individuals (459 patients with coronary artery disease or myocardial infarction and 458 healthy individuals). We used matched genetic and expression data from this study from all 758 individuals to compute an independent set of CD14+ monocyte expression weights. A total of 3163 genes were modelled with significant *cis*-heritable expression. We replicated the TWAS using the Kunkle summary statistics,^8^ so that we could compare our results at baseline with the TWAS results from CD14+ monocytes derived from the Fairfax et al data. Significant TWAS results for CTS and Fairfax CD14+ are shown in Table 2, with complete results of the CTS TWAS in Supplementary Table 2. The mirrored manhattan plot (Supplementary Fig. 5) shows that *LACTB2, SPI1, MYBPC3* and *MS4A4A* and *MS4A6A* are TWAS significant in the CTS TWAS, with the same direction of effect as in the Fairfax TWAS. Three genes, *TNFRSF21, EPHA1* and *CLPTM1* are TWAS significant only in the CTS TWAS. The relationship between the CTS and Fairfax TWAS results is shown graphically in Supplementary Fig. 6.

**Table 2:** Comparison of transcriptome-wide association study (TWAS) test statistics for significantly associated genes for Alzheimer's disease in two independent CD14+ monocytes samples

Gene	Chromosome	Gene start	Gene end	FAIRFAX CD14 TWAS.Z	CTS TWAS.Z	FAIRFAX CD14 TWAS.P	CTS TWAS.P
*TNFRSF21*	6	47199268	47277641	NA	−5.04852	NA	4.45E−07
*EPHA1*	7	143087382	143105985	NA	−4.853282	NA	1.21E−06
***LACTB2***	8	71547553	71581409	4.305	4.3956	1.67E−05	1.10E−05
*PTK2B*	8	27168999	27316903	5.485	NA	4.14E−08	NA
MS4A4A**	11	60048014	60076445	6.683	4.966	2.33E−11	6.84E−07
MS4A6A*	11	59939081	59952139	6.431	7.9397	1.27E−10	2.03E−15
***MYBPC3***	11	47352957	47374253	5.204	5.1329	1.95E−07	2.85E−07
SPI1***	11	47376411	47400127	6.397	6.5571	1.58E−10	5.48E−11
*STX3*	11	59480929	59573354	−4.898	NA	9.66E−07	NA
*CLPTM1*	19	45457842	45496599	NA	−8.36565	NA	5.98E−17

Genes showing TWAS significant associations (*P* < 0.05 after Bonferroni correction for the number of genes for which TWAS was performed in each tissue). TWAS.P values shown are not corrected for multiple testing. CD14_TWAS.Z and CTS_TWAS.Z denote the gene-level TWAS *Z*-score using the monocyte expression data from CD14+ cells from Fairfax et al.^24^ and the CTS,^30^ respectively, together with Kunkle 2019^8^ summary statistics. TWAS significant genes that replicate between the CD14+ monocytes from the Fairfax and CTS TWAS are shown in bold.

* Chromosome 11 genes that pass conditional analysis in CTS only.

** Chromosome 11 genes that pass conditional analysis in FAIRFAX only.

*** Chromosome 11 genes that pass conditional analysis in both FAIRFAX and CTS CD14+ monocytes.

### Comparison with TWAS from independent summary statistics

We replicated the TWAS using independent summary statistics from UK Biobank GWAS.^33^ This study used Alzheimer’s disease diagnosis based on a proxy by parental diagnosis design. Overall, the correlation in gene-wide *Z* between the clinical Alzheimer’s disease^8^ and Alzheimer’s disease-proxy analyses^33^ was modest (*r* ∼0.2), although statistically significant (CD14: *P* = 2.2 × 10^−16^, IFN: *P* = 4.9 × 10^−14^, LPS2: *P* = 2.2 × 10^−16^, LPS24: *P* = 5.6 × 10^−16^) (Supplementary Fig. 4). Table 1 shows the Marioni TWAS *Z*-scores and *P*-values for the associated genes reaching statistical significance (after Bonferroni correction) in the Kunkle sample. It is notable that the sign of the *Z*-score which indicates the direction of effect, was identical in both samples for all genes. Of the 24 associations in Table 1, 15 achieved nominal significance (*P* < 0.05) in the Marioni dataset. Three genes in Alzheimer’s disease risk loci from GWAS: *PTK2B*, *MS4A6E* and *PVR* gave a significant *P*-value (after Bonferroni correction) in the Marioni dataset. The novel genes, *PLIN2* and *LACTB2*, showed reduced evidence for TWAS association in the Marioni dataset, although *LACTB2* showed a nominally significant association in LPS24 (Table 1; Supplementary Fig. 4).

### Comparison of TWAS from monocytes with TWAS from other tissues

**Figure 3:**
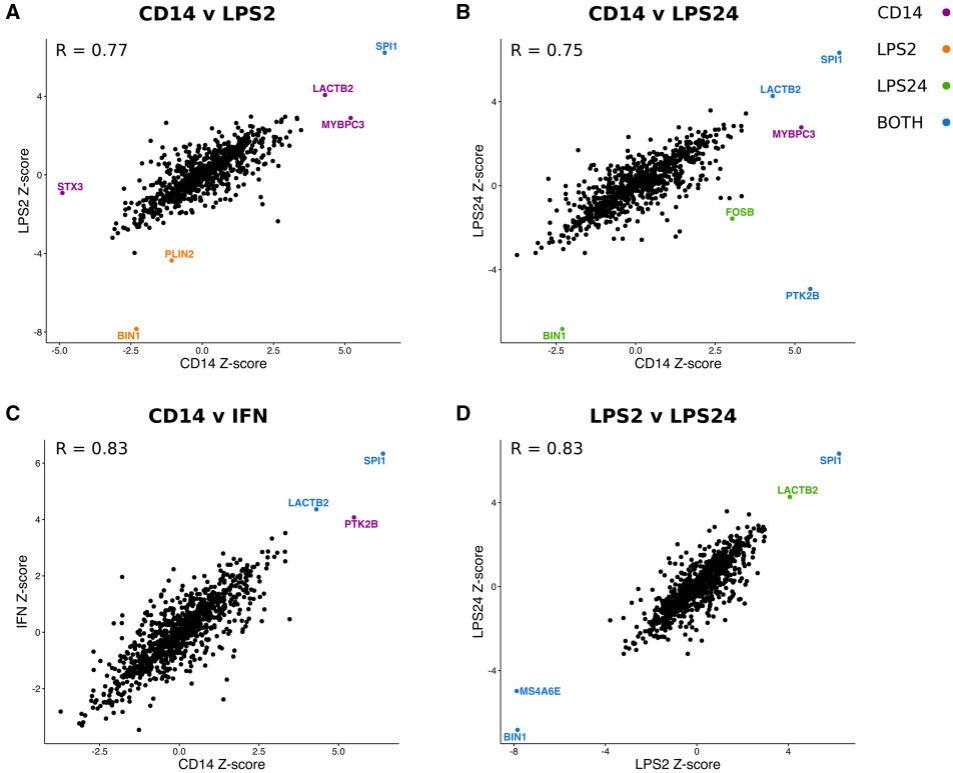
**Correlation plots of *Z*-scores from TWAS in monocytes. Genes are colour-coded according to their TWAS significance in cells each monocyte lineage: naïve CD14 = purple; LPS2-induced = orange; LPS24_induced = green or in both cell types (blue).** The increase in expression of SPI1 associated with Alzheimer’s disease is present in all of the monocyte lineages and that of LACTB2 in naïve CD14, LPS24 induced and IFN induced cells. R denotes the Pearson correlation coefficient between the *Z*-scores in each case.

**Figure 4:**
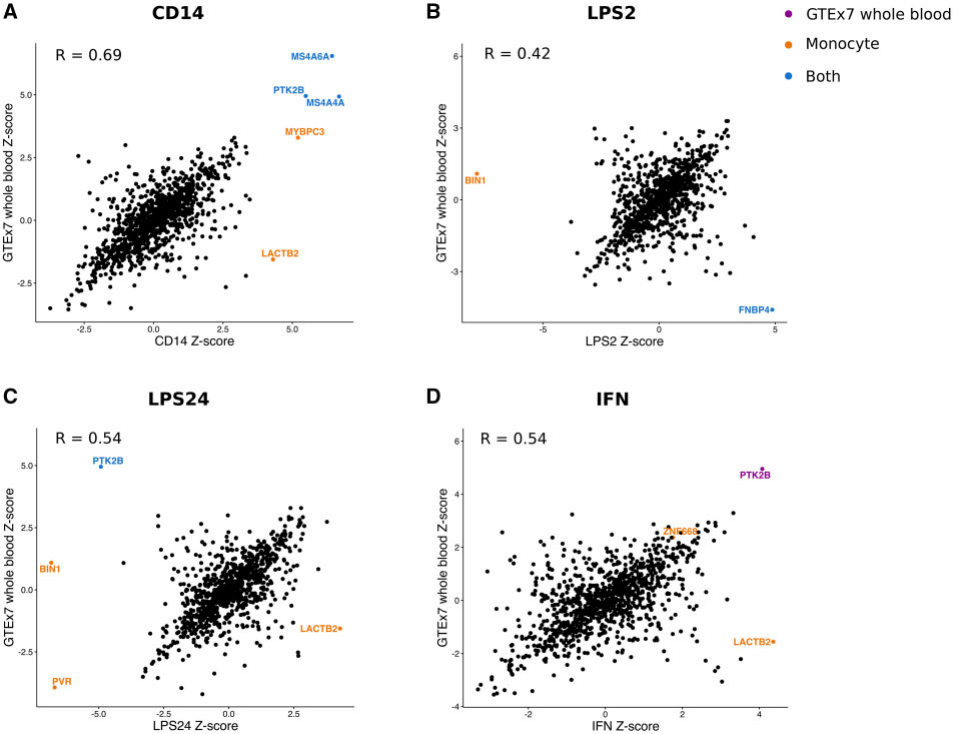
**Correlation plots of *Z*-scores from TWAS in monocytes and whole blood. Genes are colour-coded according to their TWAS significance in cells in the monocyte lineage (orange), GTEx7 whole blood (purple) or both (blue).** The increase in expression of LACTB2 associated with Alzheimer’s disease is only present in the monocyte lineage. R denotes the Pearson correlation coefficient between the *Z*-scores in each case.

To test whether Alzheimer’s disease-associated changes in gene expression were specific to monocytes, or whether they act across tissues, we performed TWAS analyses on the Kunkle et al.^8^ GWAS summary statistics using published expression weights from the GTEx (Genotype-Tissue expression) consortium,^37^ the young Finns study, whole blood (YFS),^38^ the Netherlands twin register peripheral blood (NTR),^39^ and the CommonMind Consortium (URL.15) (Supplementary Table 5). Correlation plots of the *Z*-scores from the TWAS analysis showed a high correlation between *Z* scores of the genes for which expression weights were computed in multiple stimulation conditions of the monocytes (*R* > 0.75 in each case) (Fig. 3). Correlation plots of the *Z*-scores from the TWAS analysis for each monocyte stimulation condition and GTEx whole blood (Fig. 4) show that *LACTB2* was TWAS significant specifically in monocytes. In contrast, *PTK2B* was TWAS significant in CD14+ cells, LPS24 cells and whole blood (*r* = 0.4–0.7, Fig. 4 and Supplementary Fig. 1). Similar correlation plots of the *Z*-scores from the TWAS analysis are shown for each monocyte stimulation condition versus, peripheral blood (NTR) (*r* = 0.3–0.6, Supplementary Fig. 2) and CMC brain, dorsolateral prefrontal cortex (DLPFC) (*r* = 0.3–0.4, Supplementary Fig. 3). We calculated the correlation coefficients between all combinations of the Alzheimer’s disease relevant tissues [GTEx and CMC Brain, GTEx and METSIM Adipose, GTEx and YFS whole blood, NTR peripheral blood and each of the monocyte stimulation conditions (Supplementary Table 6)]. These results show that the largest correlation in *Z*-scores is between the monocyte conditions, then between the monocyte conditions and blood. The correlation between the monocyte conditions and the brain tissues tested in all cases is low (*r* ≤ 0.5). The *P* value and number of genes in each pairwise correlation is summarized in Supplementary Table 5.

### TWAS significant genes in Alzheimer’s disease risk loci from GWAS

For the MS4A4 locus and *PTK2B* and *PVR*, where the TWAS signal and direction effect replicated using independent summary statistics from the UK Biobank GWAS, we present the results below. For the remaining genes in Alzheimer’s disease risk loci from GWAS, not replicated, we discuss the results in the Supplementary material.

### PTK2B

We show that there is an Alzheimer’s disease-associated increase in expression of *PTK2B* in CD14+ monocytes (Fairfax), whole blood and peripheral blood (GTEx7 and YFS, respectively). However, the TWAS result in Fairfax CD14+ monocytes did not replicate in the TWAS using expression weights derived from CTS study data as *PTK2B* was not modelled with significant *cis*-heritable expression. Although we see an Alzheimer’s disease-associated decrease in expression of *PTK2B* in LPS-induced monocytes (24 h), this is not replicated when using the Marioni summary statistics and we do not see an association in IFN-induced monocytes. This association is not seen in other GTEx tissues, e.g. in Brain Cerebellum suggesting that the association of this gene with Alzheimer’s disease is specific to blood, but it is not associated with monocyte induction.

### PVR

We have shown that a decrease in expression of *PVR* is associated with Alzheimer’s disease in LPS-induced monocytes (24 h) and this result replicated using independent summary statistics (Marioni). We also detected TWAS signals in several GTEx tissues including subcutaneous adipose, in CMC brain (DLPFC) but not in whole blood. The best eQTL (expression quantitative trait locus) SNP in Alzheimer’s disease-relevant tissues, rs10426401 is in a regulatory region in the first intron of *PVR* which could affect several transcription-factor binding sites. However, as *PVR* belongs to the *APOE* GWAS region of association, we tested whether the *PVR* prediction from TWAS is dependent on *APOE* genotype. Using logistic regression (see Materials and methods section), we show that there is a significant negative association between the number of e4 alleles and *PVR* expression and that the association of genetically predicted *PVR* expression with Alzheimer’s disease risk may be attributable to the effect of *APOE* (Supplementary Table 8).

### MS4A locus

The MS4A region is a genome-wide significant Alzheimer’s disease risk locus. Multiple genes in this locus are in high linkage disequilibrium (LD). We detected a significant association between an increase in expression of *MS4A4A* and *MS4A6A* and Alzheimer’s disease risk in both the Fairfax and in the CTS TWAS in CD14+ monocytes and the direction of effect is the same in both TWAS. After conditional analysis of the Fairfax TWAS results, we detected an association between an increase in expression of *MS4A4A* in naïve CD14+ monocytes and Alzheimer’s disease. However, the results of the conditional analysis for these two genes in CD14+ monocytes from the CTS study and the Fairfax study conflict. (Supplementary Table 4 and Supplementary material—Conditional analysis plots). In the Fairfax CD14+ TWAS, *MS4A4A* appears to be responsible for the TWAS associations at the locus, whereas in the CTS CD14+ TWAS, the signal appears to be driven by *MS4A6A*. In addition, we identified a statistically significant association between a decrease in *MS4A4E* gene expression and Alzheimer’s disease in the LPS-induced monocytes (2 and 24 h). This result was replicated in LPS2 cells using the Marioni summary statistics. We did not detect an association between changes in expression of *MS4A6E* and Alzheimer’s disease in any of the GTEx tissues, YFS or NTR blood, which contradicts a previous report of an eQTL effect (an increase in expression) in whole blood.^40^ However, the decrease in gene expression associated with Alzheimer’s disease that we identified at *MS4A6E* using TWAS may be specific to LPS-induced monocytes.

### Two novel Alzheimer’s disease-associated genes, LACTB2 and PLIN2

We detected a significant association between Alzheimer’s disease risk and an increase in expression of *LACTB2* in CD14+ monocytes in both the Fairfax and in the CTS TWAS. In addition, *LACTB2* was TWAS significant in LPS (LPS24) and IFN-induced monocytes (Fig. 3). *LACTB2* was modelled with significant *cis*-heritable expression in 27 GTEx7 tissues which included whole blood, seven brain tissues, YFS blood and NTR blood. However, *LACTB2* was not TWAS significant in any of these tissues (Supplementary Tables 5 and 7).

*PLIN2* was modelled with significant *cis*-heritable expression in YFS blood and NTR blood, 2 GTEx7 cell lines, EBV-transformed lymphocytes and transformed fibroblasts, but not in any of the GTEx7 tissues. However, *PLIN2* was only TWAS significant in LPS2-induced monocytes.

### Functional analysis of the top eQTL variants in LACTB2 and PLIN2 in monocytes

In the TWAS analyses of *LACTB2*, the variant most significantly associated with Alzheimer’s disease risk from GWAS, rs7830986, was also the variant most significantly associated with an increase in gene expression in induced monocytes (LPS24 and IFN; Supplementary Table 2). Previously, this variant was shown to be a significant eQTL (*P* = 1.7e−81) in monocytes.^30^ From TWAS, we detected an association between an increase in *LACTB2* expression and Alzheimer’s disease, which is in agreement with the eQTL study. rs7830986 is a synonymous variant in the *LACTB2* protein-coding gene and it is also within the lncRNA, *LACTB2-AS1* and an enhancer region. This SNP is likely to be involved in the regulation of *LACTB2* expression though the lncRNA or by affecting one of the transcription-factor binding sites in the enhancer region. The most significantly associated eQTL variant in naïve CD14+ monocytes, rs13271014 is an intron variant in a regulatory region that overlaps the anti-sense lncRNA *LACTB2_AS1*.

The most significantly associated eQTL variant in *PLIN2* in LPS2 induced monocytes, rs10757004 is an intron variant in the *SAXO1* gene. This SNP is not in a regulatory region of the genome.

## Discussion

Previously, CD14+ monocytes from a different dataset (BLUEPRINT)^41^ have been used in a multi-tissue TWAS study of Alzheimer’s disease,^42^ alongside expression data from GTEx, using a smaller GWAS^43^ than that used here. They report associations with *MS4A4A* and *MS4A6A*, consistent with our results showing TWAS associations with these genes across a range of tissues (Supplementary Table 5). Monocyte expression from the Fairfax and CTS datasets has been studied previously in the context of Alzheimer’s disease,^44^ also using the Lambert et al. GWAS samples. However, that study used a slightly different phenotype (age at onset), different analysis techniques to relate expression to GWAS signal, and restricted the analyses to CD14+ monocytes. Some of the associations reported here were also observed in Huang et al.^44^ notably those with *SPI1*, *MS4A4A*, *MS4A6A*, but others are novel.

The extent to which patterns of TWAS associations are consistent across monocytes, brain and blood, has not been studied previously. In the work presented here, we used the latest Alzheimer’s disease GWAS,^8^ two independent sets of CD14+ monocyte data (Fairfax and CTS)^24^^,^^30^ and data from LPS and IFN-induced monocytes^24^ to compute TWAS weights. Since the CTS study only contains data from CD14+ monocytes, it was not possible to replicate the TWAS associations seen in the Fairfax monocyte data after induction with LPS and IFN, which is a limitation of this study.

We found that the TWAS results from the four Fairfax monocyte conditions were strongly correlated with each other and they were also correlated with those from whole blood and peripheral blood. Correlation with CMC brain (DLPFC) was less strong, a pattern also observed for the GTEx brain tissues.

We detected an association between changes in gene expression in both naïve and induced CD14+ monocytes and Alzheimer’s disease. For three genes in Alzheimer’s disease risk loci from GWAS, *PVR*, *PTK2B* and *MS4A4E*, the TWAS results replicated using independent summary statistics from Marioni et al.^33^ However, for the remaining genes with statistically independent TWAS signals that are in Alzheimer’s disease risk loci, A*POE*, *BIN1, LACTB2, MS4A6E, PLIN2/ADRP* and *SPI1*, we note that the results did not replicate. This may be explained by the fact that these summary statistics were derived from UK biobank data using a proxy-phenotype design. The UK biobank data included family history information (parent or first-degree relative with Alzheimer’s disease or dementia) as a proxy-phenotype for the participants and this study is based on self-reporting of any type of dementia, which may contribute to ‘noise’ in the summary statistics. As a consequence, the effect sizes from the Marioni study are systematically smaller than those from the Kunkle study. Our criterion for replication (Bonferroni significance for the total number of genes tested for that tissue in both the Kunkle and Marioni datasets) is also quite stringent. Thus, further study of the role of expression on Alzheimer’s disease risk is warranted for the genes mentioned above, even if they do not meet formal criteria for replication.

*PTK2B* encodes a cytoplasmic protein that is a member of the FAK subfamily of protein tyrosine kinases which are important in adhesion signalling and cell motility and it is highly expressed in monocytes and in the brain. We detect an association between an increase in *PTK2B* expression and Alzheimer’s disease specifically in monocytes and blood, suggesting that the association in Alzheimer’s disease is specific to blood. The most significantly associated eQTL SNP, rs17057043 in CD14+ monocytes, peripheral (NTR) and whole (YFS) blood and in LPS-induced monocytes is located in intron 5 of *PTK2B*. Previously this SNP has been reported to be an eQTL in monocytes.^30^^,^^45^ This variant affects the binding of IRF1, a transcription factor that regulates the innate and acquired immune response and it is involved in some of the cytokine responses to LPS.^46^ Interestingly, it has been shown previously that other genes in Alzheimer’s disease risk loci are regulated by IRF1.^47^ However, we note that although the *PTK2B* signal replicates in Fairfax CD14+ monocytes using the Marioni summary statistics, it does not replicate in the CTS monocyte TWAS.

PVR/CD155 was first identified as the receptor for polio virus, but it has been shown to have many biological roles.^48^ It is important in the immune response, regulating cell-mediated immunity by promoting monocyte trans-endothelial migration (TEM).^49^*PVR* has been associated with Alzheimer’s disease previously in an Alzheimer’s disease-by-proxy meta-analysis.^33^ The best eQTL SNP in Alzheimer’s disease-relevant tissues, rs10426401 is in a regulatory region in the first intron of *PVR* and could affect several transcription-factor binding sites. We have shown that genetically predicted *PVR* expression is correlated with the number of *APOE* e4 alleles, and that the TWAS association with Alzheimer’s disease risk may therefore be due to *APOE* effects.

The MS4A locus is a complex locus containing many genes which are in are in high LD. From Alzheimer’s disease-GWAS, rs4938933 which is upstream of *MS4A4*^50^ and rs610932 which is in the promotor of *MS4A6A*^51^ have been reported as genome-wide significant and recently, both MS4A4A and MS4A6A have been shown to modulate TREM2, a component of a transmembrane receptor-signalling complex that is important in microglial activation and function.^52^

The most significantly associated eQTL SNP in Fairfax CD14+ monocytes in our study was rs6591559 which has been reported previously as an eQTL in monocytes.^45^

This SNP is upstream of *MS4A4A* in the intergenic region between the *MS4A4A* and *MS4A4E.* In the CTS CD14+ monocyte TWAS, rs610932 is the most significantly associated eQTL SNP, which is in the promoter of *MS4A6A*. This association at *MS4A4A* and the direction of effect has been shown in GTEx7 whole blood (rs573122) and adipose tissue (rs12421663) and thyroid (rs600550), (Supplementary Table 5).^8^ We note that Huang et al.^44^ also have shown *cis*-eQTL associations with expression in both *MS4A4A* and *MS4A6A* using the CTS and Fairfax monocyte data but applying a different TWAS method. They showed that the minor allele of the most significantly associated SNP at this locus, rs7930318, located in the intergenic region between *MS4A4A* and *MS4A4E*, was associated with both delayed age at onset and lower expression of *MS4A4A* in monocytes. This agrees with our observation of increased *MS4A4A* expression being associated with Alzheimer’s disease risk.

In our study in LPS-induced monocytes the most significantly associated eQTL SNP is rs11824734, which is also upstream of *MS4A4A*. Both of the SNPs in this locus are in regulatory regions, but they are not in a known transcription factor binding site.

Our TWAS analyses provide evidence that expression of *MS4A4A* and *MS4A6A* in CD14+ monocytes is relevant to Alzheimer’s disease risk, although conditional analyses are unable to conclusively localise the signal to either of these genes. Nevertheless, the evidence taken together points to Alzheimer’s disease-associated variants within the intergenic region between *MS4A4A* and *MS4A4E* affecting the regulation of these genes. Using TWAS we have filtered the associations between the variants in the MS4 locus and Alzheimer’s disease in monocytes. We have shown an association between an increase in expression of *MS4A4A* and *MS4A6A* in naïve CD14+ monocytes and Alzheimer’s disease, whereas, after induction with in LPS-induced monocytes, a decrease in gene expression of *MS4A4E* is associated with Alzheimer’s disease.

For two genes not previously associated with Alzheimer’s disease from GWAS, *LACTB2* and *PLIN2*, we detected a significant TWAS association signal in the Kunkle et al. dataset. This is interesting because in gene-wide analysis of the Kunkle et al. summary statistics using MAGMA,^35^*PLIN2* and *LACTB2* are not significant (*P* = 5.99 × 10^−4^ for *LACTB2*, *P* = 6.72 × 10^−2^ for *PLIN2*). Furthermore, *LACTB2* showed a nominally significant TWAS association in LPS24 in the independent Marioni et al. dataset. The association between a decrease in expression of *PLIN2* and Alzheimer’s disease was detected exclusively in LPS2 induced monocytes. We note that the TWAS signal for *PLIN2* did not replicate using independent summary statistics and after conditional analysis. Therefore, the evidence that the association with Alzheimer’s disease is due to a change in gene expression is weak. However, this gene is TWAS-significant and it is interesting in the context of monocyte induction by LPS.

*PLIN2* encodes Perilipin-2^53^ which is one of the most abundant proteins in intracellular neutral lipid storage droplets (LSDs). LSDs contain a core of neutral lipids which are encapsulated in a monolayer of phospholipids and proteins. The lipids stored in LSDs are used in metabolism, membrane synthesis and cholesterol homeostasis and they are thought to be important in immune responses in myeloid cells.^54^ LSDs are mobile within the cytoplasm and can move to associate with the endoplasmic reticulum (ER) and mitochondria. Many genes in Alzheimer’s disease risk loci from GWAS are involved in lipid metabolism, e.g. *APOE*,^55^*ABCA7*,^56^*CLU/APOJ*,^57^ and *RORA*^58^ and lipid metabolism pathways have been identified as key in Alzheimer’s disease.^7^ Recently it has been shown that perilipins are regulated by peroxisome proliferator-activated receptor gamma (PPAR-gamma) which, when inhibited results in the down-regulation of *PLIN2*, affecting LSD formation.^59^*RORA* encodes nuclear receptor ROR-alpha, which is a regulator of lipid homeostasis through PPAR-gamma.^60^ Hence, using TWAS, we have connected two genes involved in lipid metabolism through PPAR-gamma.

The association between an increase in expression of *LACTB2* and Alzheimer’s disease was detected in monocytes at baseline and after induction with IFN or LPS, suggesting that the association between *LACTB2* and Alzheimer’s disease is not specific to monocyte induction. *LACTB2* is widely expressed^61^ and it is a component of the core oligodendrocyte gene set (COLGS).^62^*LACTB2* encodes a mitochondrial endoribonuclease that cleaves ssRNA but not dsRNA or ssDNA. LACTB2 is essential for mitochondrial function and cell viability and its overexpression in cultured cells causes a reduction in many mitochondrial transcripts.^63^ Mitochondrial dysfunction has been implicated in Alzheimer's disease and across several other neurodegenerative disorders such as Parkinson's and Huntington's disease^64^ and across neuropsychiatric disorders such as bipolar disorder, depression and Schizophrenia.^65^ Mitochondrial dysfunction results in defects in calcium signalling and apoptotic pathways, ATP-depletion which affects oxidative phosphorylation and in the depletion of mitochondrial DNA. Malfunction of mitochondrial processes implicated specifically in Alzheimer’s disease include oxidative damage^66^ and accumulation of APP on mitochondrial membranes.^67^ More recently it has been shown that mitochondria are important in immune cell regulation; influencing immune cell metabolism, differentiation, activation of the inflammatory response and regulating transcription.^68^ Variants in mitochondrial DNA (mtDNA) have been associated with Alzheimer’s disease,^69^ but many of the processes that affect mitochondrial function, involve nuclear-encoded proteins,^70^ so it is more likely that variants in nuclear genes that affect mitochondrial function will affect Alzheimer’s disease pathology. However, it is also possible that mitochondrial dysfunction may itself lead to disease pathology. LACTB2 may have a role in regulating mitochondrial mRNA turnover, and since mitochondria regulate the immune response, defects in *LACTB2* gene function may affect the inflammatory response.

## Conclusion

We have used TWAS to dissect the functional variants associated with Alzheimer’s disease in the context of monocyte differentiation in response to immune stimuli. We have shown an association between changes in gene expression in naïve and induced CD14+ monocytes and Alzheimer’s disease in seven genes in known Alzheimer’s disease loci, three of which (*PVR, PTK2B* and *MS4A6E*) replicated in TWAS using independent summary statistics. We also detected TWAS signals in two genes, *LACTB2* and *PLIN2* that have not been associated with Alzheimer’s disease previously.

## Supplementary material

Supplementary material is available at *Brain Communications* online.

## Supplementary Material

fcab083_Supplementary_Data

## Abbreviations

CD14+: = Naïve monocytes expressing CD14
CMC =: CommonMind Consortium
CTCF =: CCCTC-binding factor
CTS =: Cardiogenics transcriptomic study
DLPFC =: dorsolateral prefrontal cortex
EOAD =: early-onset AD
eQTL =: expression quantitative trait locus
GTEx =: genotype-tissue expression
GWAS =: genome-wide association studies
HWE =: Hardy Weinberg Equilibrium
IFN =: CD14+ stimulated with interferon-gamma
LOAD =: late onset AD
LPS2 =: CD14+ cells stimulated with lipopolysaccharide for 2 h
LPS24 =: CD14+ cells stimulated with lipopolysaccharide for 24 h
LSDs =: intracellular neutral lipid storage droplets
MAF =: minor allele frequency
miRNA =: micro-RNA
mtDNA =: mitochondrial DNA
NTR =: Netherlands twin register peripheral blood
SNP =: single nucleotide polymorphism
TWAS =: transcriptome-wide association studies
YFS =: Young Finns study, whole blood
