# Supplementary material for: Defining functional variants associated with Alzheimer’s disease in the induced immune response
Source: Brain Commun. 2021 Apr 19;3(2):fcab083. doi: 10.1093/braincomms/fcab083 (PMC8087896; doi:10.1093/braincomms/fcab083)
Supplement: fcab083_Supplementary_Data [file fcab083_supplementary_data.zip › Supplementary_Material.pdf]

Supplementary Material

TWAS-significant genes located in known AD risk loci from GWAS *3*

APOE 3

BIN1 3

SPI1 3

Supplementary Figures **5**

Supplementary Fig 1. Correlation plots of Z-scores from TWAS in monocytes and YFS blood. 5

Supplementary Fig 2. Correlation plots of Z-scores from TWAS in monocytes and NTR blood 6

Supplementary Fig 3. Correlation plots of Z-scores from TWAS in monocytes and CMC dorsolateral pre-fontal cortex (DLPFC). 7

Supplementary Fig 4. Correlation plots of Z-scores from TWAS using Kunkle and Marioni summary statistics. 8

Supplementary Fig 5. Mirrored manhattan plots of TWAS results in Cardiogenics Transcriptomic study (CTS). 9

Supplementary Fig 6. Correlation plots of Z-scores from TWAS using gene expression weights from the Cardiogenics Transcriptomic study (CTS) and the Fairfax study. 10

Supplementary Tables. **11**

Supplementary Table 1. TWAS significant genes in the Kunkle and Marioni analysis for each monocyte cell type. 11

Supplementary Table 2 (excel sheet ST2). Results of TWAS analysis from monocytes. 11

Supplementary Table 3. 12

Supplementary Table 4. Summary of the TWAS significant genes across the monocyte cell types. 13

Supplementary Table 5 (excel sheet ST5). Results of TWAS analysis from 52 tissues. 14

Supplementary Table 6 (excel sheet ST6). Correlation of TWAS Z-scores between monocytes and AD-relevant tissues. 14

Supplementary Table 7. Transcriptome-wide association study (TWAS) test statistics for genes in blood and brain. 15

Supplementary Table 8. PVR prediction is dependent on APOE genotype. 16

Conditional analysis plots **17**

1. PTK2B locus *17*

2. LACTB2 locus *18*

3. SPI1 locus *19*

4A. MS4A4 locus *20*

4B. MS4A4 locus *21*

5. BIN1 locus *22*

6. PLIN1 locus *23*

7. MS4A6E locus *24*

8. APOE locus *25*

9. PVR locus *26*

Supplementary references. **27**

TWAS-significant genes located in known AD risk loci from GWAS

APOE

The *APOE* gene encodes apolipoprotein E which binds fat to form lipoproteins.
Its association with AD is well established through GWAS[1] and being a carrier of the E4 allele of the *APOE* gene is one of the strongest risk factors for AD.[2] We have shown that an increase in expression of *APOE* is associated with AD in LPS induced monocytes (2 hours) but this is not evident in monocytes induced with LPS for 24 hours. However, the same eQTL and direction of change expression is seen in GTEx lung tissue. The most significantly associated eQTL SNP, rs5157 is an intron variant of *APOC4* that is located in a regulatory region upstream of the *APOC2* gene.

BIN1

We have shown that a decrease in expression of *BIN1* is associated with AD in LPS induced monocytes (2 and 24 hours), but not in naive CD14+ or in IFN-induced monocytes.
The most significantly associated eQTL SNP, rs6710467 is an intergenic variant upstream of *BIN1* that has been associated with acute myeloid leukaemia.[3]
A decrease in expression of this gene is also associated with AD in ten of the GTEx7 tissues, using TWAS, but only two of these are AD-relevant; Brain Cerebellum and Brain Cerebellar Hemisphere. We note that previously published eQTL studies show similar results to our TWAS analysis for the association between a change in expression in *BIN1* and rs6710467 in monocytes,[4] whole blood and cerebellum.[5]

SPI1

In all four monocytes, we detected a monocyte-specific association between an increase in the expression of *SPI1* and AD. These associations were not seen in whole blood or peripheral blood, they are specific to monocytes. In naive CD14+ cells, IFN and LPS24 induced cells, the most significantly associated eQTL SNP was rs10838698. This SNP has been reported to be significantly associated with *SPI1* expression in monocytes and macrophages.[6] However, the intronic variant, rs755553 is the most significantly associated

eQTL SNP in LPS2 cells in this analysis. This SNP is in a regulatory region of the *SLC39A13* gene.

The *SPI1* gene encodes PU.1, an ETS-domain transcription factor that activates gene expression during myeloid and B-lymphoid cell development[7] and in the CNS it controls the development of microglia. It is a master regulator that regulates several AD-risk genes.[6]
Our analysis using AD summary statistics from Kunkle *et al* is in agreement with previous work and expands the association of a change in *SPI1* expression to both naive and induced monocytes.

Supplementary Figures

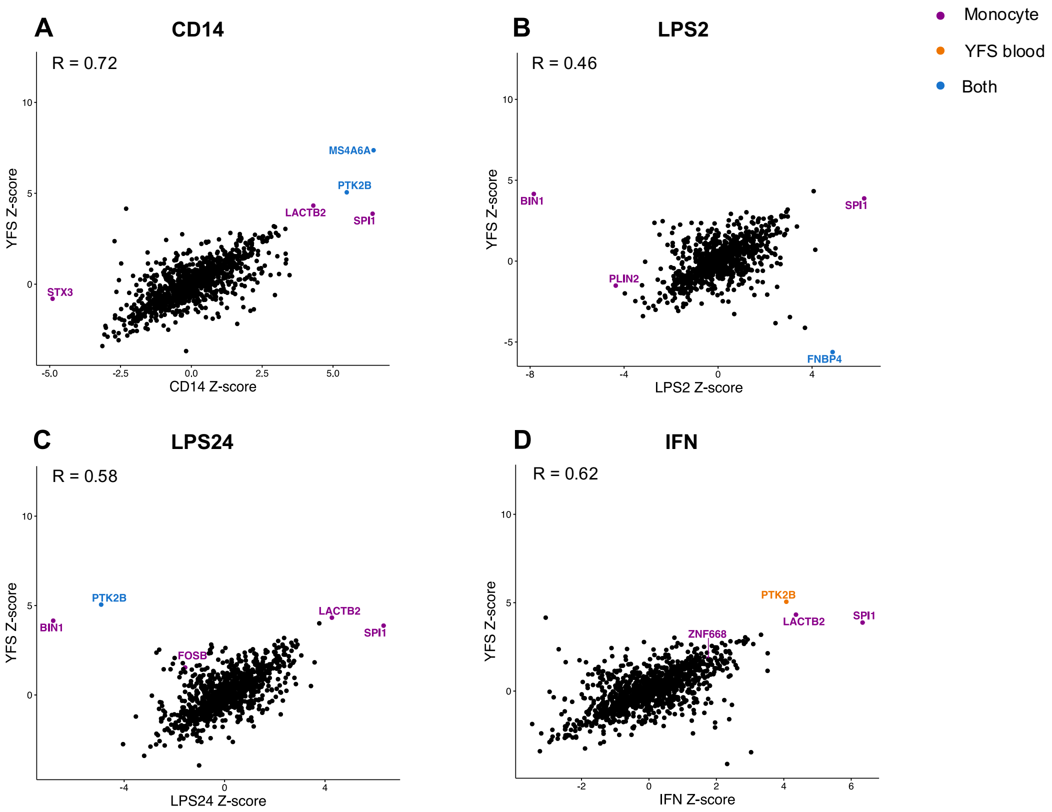

Supplementary Fig 1. Correlation plots of Z-scores from TWAS in monocytes and YFS blood. Genes are colour-coded according to their TWAS significance in monocyte lineages (purple), YFS blood (orange) or in both cell types (blue). R denotes the Pearson correlation co-efficient between the Z-scores in each case.

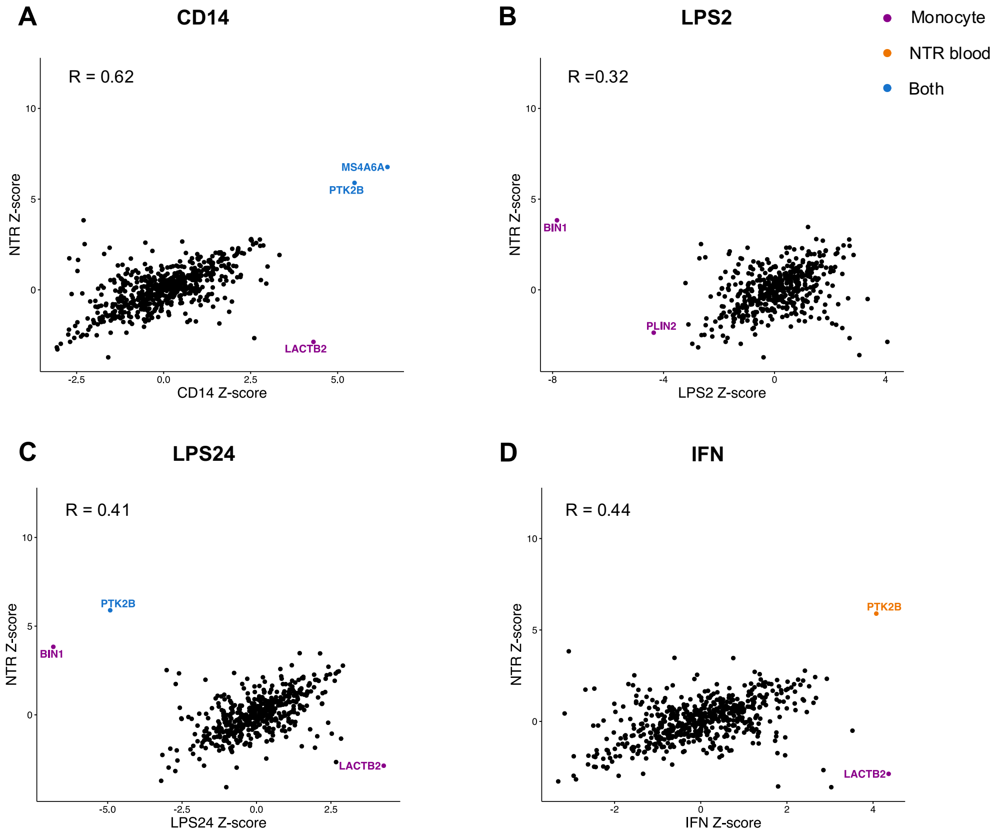

Supplementary Fig 2. Correlation plots of Z-scores from TWAS in monocytes and NTR blood. Genes are colour-coded according to their TWAS significance in monocytes (purple,) NTR blood (orange) or in both cell types (blue). R denotes the Pearson correlation co-efficient between the Z-scores in each case.

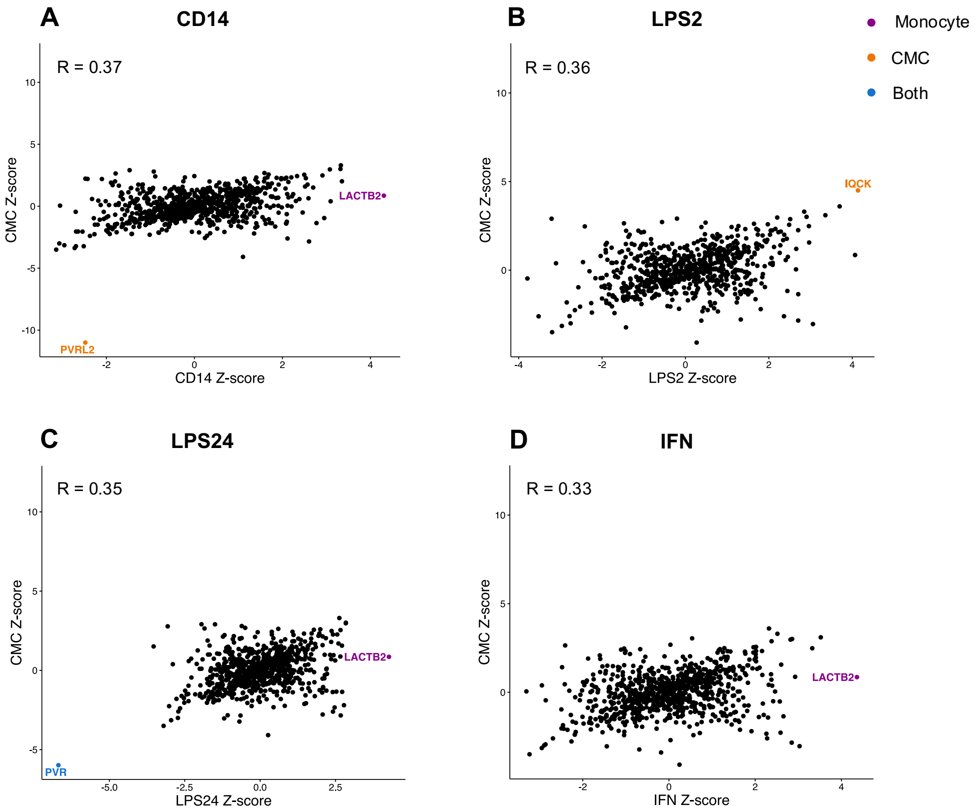

Supplementary Fig 3. Correlation plots of Z-scores from TWAS in monocytes and CMC dorsolateral pre-fontal cortex (DLPFC). Genes are colour-coded according to their TWAS significance in monocytes (purple), CMC - DLPFC (orange) or in both cell types (blue). R denotes the correlation co-efficient between the Z-scores in each case. R denotes the Pearson correlation co-efficient between the Z-scores in each case.

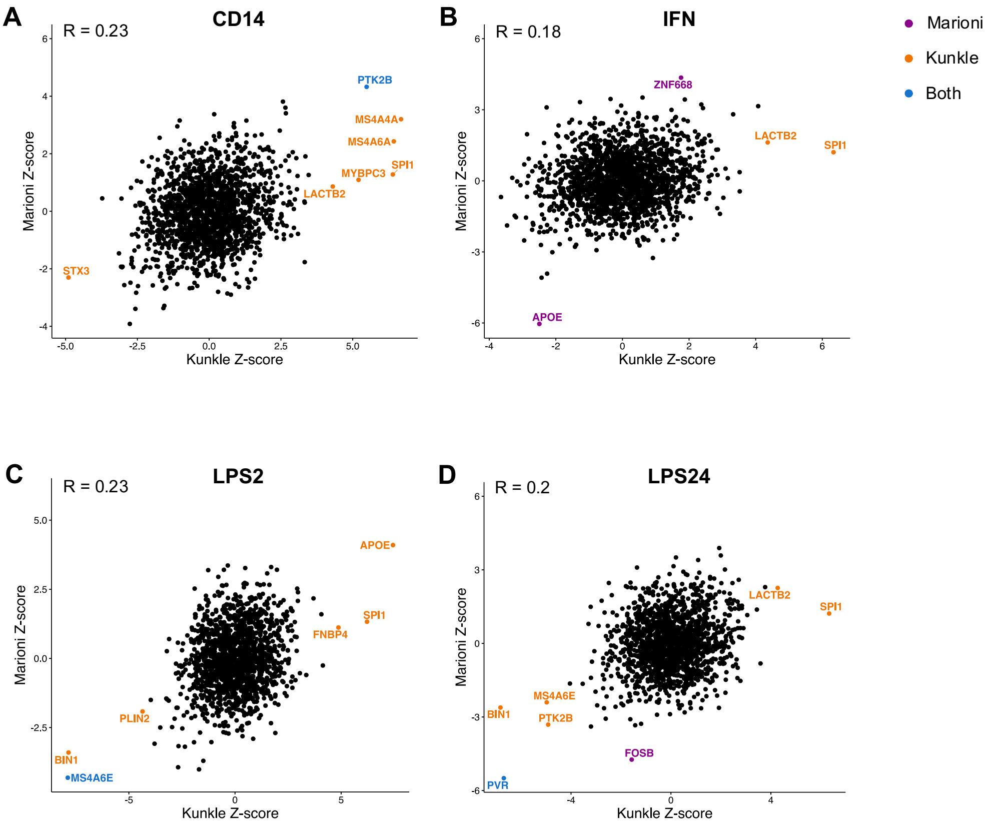

Supplementary Fig 4. Correlation plots of Z-scores from TWAS using Kunkle and Marioni summary statistics. TWAS significant genes (after Bonferroni correction) are colour-coded according to their being in the Kunkle (orange), Marioni (purple) or both (blue) summary statistics. The TWAS results for the *PTK2B*, *MS4A6E* and *PVR* genes are replicated in the Marioni TWAS in CD14+, LPS2 and LPS24 cells respectively. R denotes the Pearson correlation co-efficient between the Z-scores in each case.

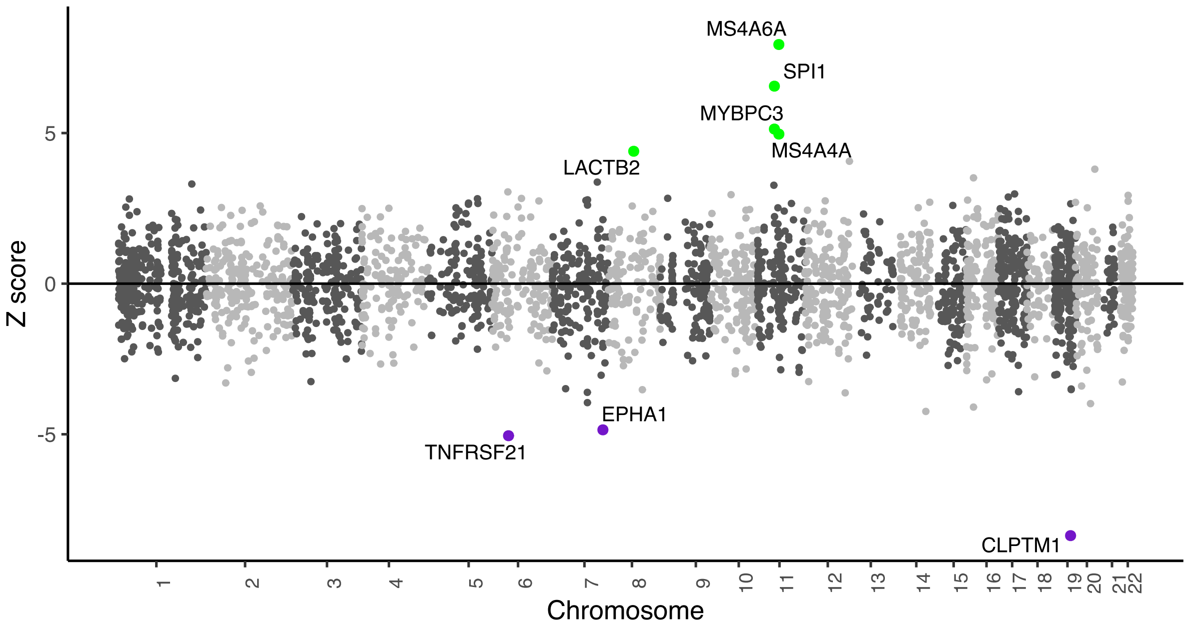

Supplementary Fig 5. Mirrored manhattan plots of TWAS results in Cardiogenics Transcriptomic study (CTS).

Genes are represented by coloured points plotted on the x axis by chromosome and genomic location. The y axis is the Z-score of the association between gene expression and Alzheimer's disease in CD14+ monocytes from the Cardiogenics Transcriptomic study (CTS). Genes that show significant association after Bonferroni correction for multiple testing are shown with green (TWAS significant in both Fairfax CD14+ and in CTS CD14+ monocytes) and purple points (TWAS significant in CTS CD14+ monocytes only) and named with black text.

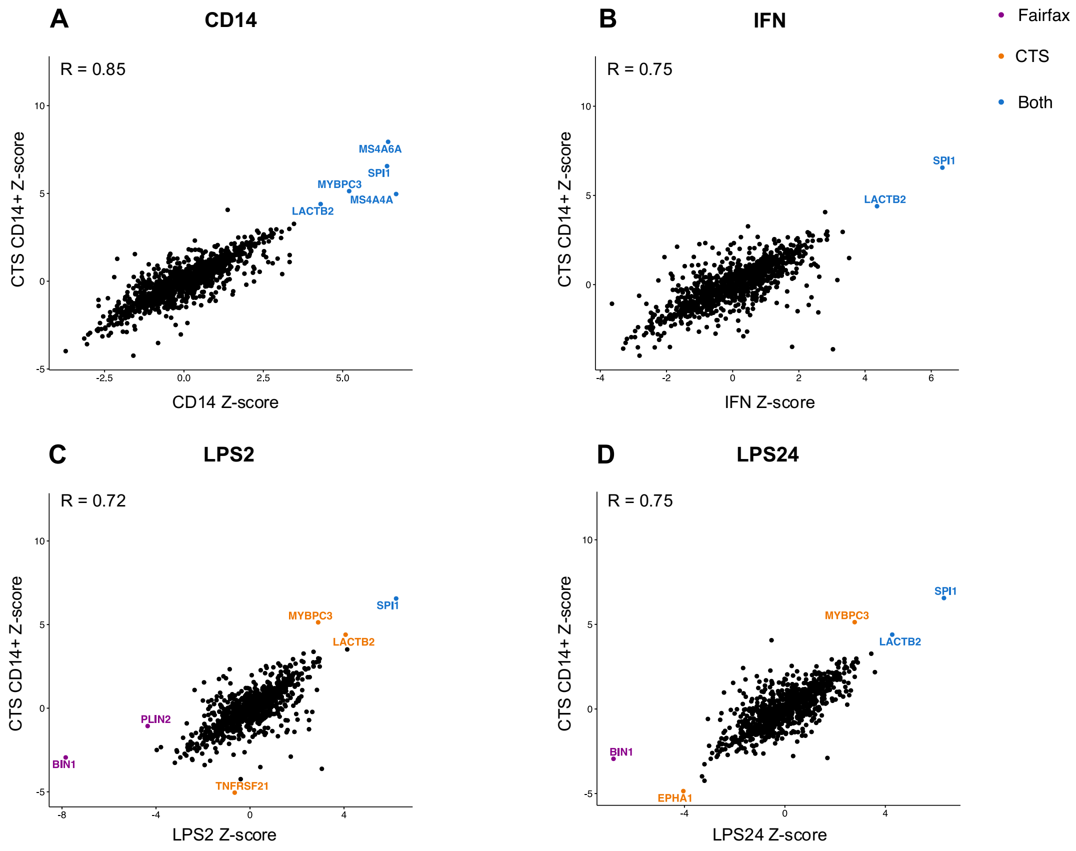

Supplementary Fig 6. Correlation plots of Z-scores from TWAS using gene expression weights from the Cardiogenics Transcriptomic study (CTS) and the Fairfax study. Genes are colour-coded according to their TWAS significance in Fairfax monocytes (CD14, LPS2, LPS24, IFN) (purple), CTS monocytes (orange) or in both (blue). R denotes the Pearson correlation co-efficient between the Z-scores in each case. *SPI1*, *MYBPC3*, *MS4A4A*, *MS4A6A* and *LACTB2* replicate between the CTS gene expression weights and the Fairfax CD14+ monocytes and the correlation between the Z scores is greatest between the Fairfax CD14+ and CTS monocytes (R= 0.85).

Supplementary Tables.

Monocyte	No. of Genes with cis-heritable expression	No. of TWAS significant genes in Kunkle	No. of TWAS significant genes in Kunkle TWAS that survive conditional analysis	No. of TWAS significant genes replicated in Marioni *
CD14	1668	7	4	1
LPS2	1559	6	5	1
LPS24	1579	6	6	1
IFN	1807	2	2	0

No. of genes with significant TWAS association after Bonferroni correction
*Marioni summary statistics = UK Biobank GWAS on parental AD - meta analysis of log-odds and SEs from maternal and paternal AD. [8]

Supplementary Table 1. TWAS significant genes in the Kunkle and Marioni analysis for each monocyte cell type. Genes with cis-heritable expression were used in the TWAS analysis for each cell type using Kunkle[5] and Marioni[8] summary statistics. Three TWAS significant genes derived using the Kunkle summary statistics were replicated in the TWAS analysis using Marioni summary statistics: *PTK2B*, *PVR*, *MS4A6E*. The numbers of TWAS significant genes in each monocyte cell type obtained using the Kunkle summary statistics before and after conditional analysis are shown.

Supplementary Table 2 (excel sheet ST2). Results of TWAS analysis from monocytes. TWAS results using Kunkle summary statistics[5] and expression weights derived from monocytes: Naive CD14+ cells (CD14), CD14+ cells induced with LPS for 2 hours (LPS2), CD14+ cells induced with LPS for 24 hours (LPS24) and IFN-induced CD14+ cells (IFN) from the Fairfax et al study[9] and an independent set of monocyte expression weights derived from the Cardiogenics Transcriptomic study (CTS).[10]

GENE/CELL TYPE	FAIRFAX CD14	CTS CD14	FAIRFAX LPS2	FAIRFAX LPS24	FAIRFAX IFN
APOE			●		○
BIN1	○	○	●	●	○
CLPTM1		●			
EPHA1		●		○	
FNBP4	○		●		
LACTB2	●	●	○	●	●
MS4A4A	●	●			
MS4A6A	●	●			
MS4A6E			●	●	
MYBPC3	●	●	○	○	
PLIN2	○	○	●		○
PTK2B	●			●	○
PVR				●	
SPI1	●	●	●	●	●
STX3	●		○		
TNFRSF21		●	○		

Supplementary Table 3.

TWAS significant genes across the monocyte cell types from both Fairfax (FAIRFAX) and the Cardiogenics transcriptomic study (CTS) for which cis-heritable expression was computed.

○ : denotes that a gene was present in the computed expression weights for a given cell type.

● : denotes that a gene was TWAS significant in the given cell type.

GENE	Chromosome	Locus	Fairfax conditional analysis	CTS conditional analysis	Monocyte	Known in AD GWAS
BIN1	2				LPS2, LPS24	[11]
PTK2B	8				CD14, LPS24	[5,12]
LACTB2*	8				CD14, LPS24, IFN	
PLIN2*	9				LPS2	
SPI1	11	CELF1			CD14, LPS2, LPS24, IFN, CTS-CD14	[6]
FNBP4	11	CELF1	dropped		LPS2	[13]
MYBPC3	11	CELF1	dropped	dropped	CD14	[6,14]
MS4A4A	11	MS4A		dropped	CD14	[15]
MS4A6E	11	MS4A			LPS2, LPS24	[13,15]
MS4A6A	11	MS4A	dropped		CD14	[16,17]
STX3	11		dropped		CD14	
APOE	19	APOE			LPS2	[1]
PVR	19	APOE			LPS24	[8]

Supplementary Table 4. Summary of the TWAS significant genes across the monocyte cell types.

13 genes were TWAS-significant across the Fairfax monocytes: CD14+ cells (CD14), CD14+ cells induced with LPS for 2 hours (LPS2), CD14+ cells induced with LPS for 24 hours (LPS24) and IFN-induced CD14+ cells (IFN). The TWAS signal for *PTK2B*, *SPI1* and *LACTB2* in Fairfax CD14+ monocytes replicated in an independent set of monocyte TWAS weights from the Cardiogenics transcriptomic study (CTS), but the signal at the MS4A4 locus remains unresolved. After conditional analysis (conditional analysis plots and Supplementary table 8) *STX3*, *MYBPC3*, *FNBP4* and *PVR* were dropped. Six genes (shown in bold) and one locus (MS4A4) remained with statistically independent TWAS signals. Two genes *LACTB2* and *PLIN2* are novel candidate genes for AD (shown with *).

Supplementary Table 5 (excel sheet ST5). Results of TWAS analysis from 52 tissues.
TWAS results using Kunkle summary statistics [5] and expression weights derived from GTEx7 tissues, CMC. BRAIN (DLPFC), METSIM.ADIPOSE, YFS.BLOOD and NTR.BLOOD.

Supplementary Table 6 (excel sheet ST6). Correlation of TWAS Z-scores between monocytes and AD-relevant tissues.
Correlation co-efficients (R2) and corresponding p-values for the correlation between the TWAS Z-scores using Kunkle summary statistics for each monocyte and relevant AD tissues: GTEx7 Brain, CMC_Brain (DLPFC) and GTEx7 whole Blood, NTR.BLOOD, YFS.BLOOD, GTEx7 Adipose and METSIM.ADIPOSE. The no. of genes in the overlap of the two variables is shown in each case.

CELL_TYPE	GENE	CHR	GENE_START	GENE_END	K_TWAS.Z	K_TWAS.P
YFS.BLOOD	*BIN1*	2	127805603	127864931	4.15	3.27E-05
NTR.BLOOD	*BIN1*	2	127805603	127864931	3.83	1.26E-04
GTEx7_Whole_Blood	*BIN1*	2	127805603	127864931	1.09	2.76E-01
**YFS.BLOOD**	***FNBP4***	**11**	**47738072**	**47788995**	**-5.62**	**1.88E-08**
**GTEx7_Whole_Blood**	***FNBP4***	**11**	**47738072**	**47788995**	**-4.60**	**4.31E-06**
YFS.BLOOD	*LACTB2*	8	71547553	71581409	4.32	1.53E-05
NTR.BLOOD	*LACTB2*	8	71547553	71581409	-2.87	4.12E-03
GTEx7_Whole_Blood	*LACTB2*	8	71547553	71581409	-1.56	1.19E-01
CMC.BRAIN	*LACTB2*	8	71547553	71581409	0.86	3.92E-01
**YFS.BLOOD**	***MS4A6A***	**11**	**59939081**	**59952139**	**7.37**	**1.73E-13**
**NTR.BLOOD**	***MS4A6A***	**11**	**59939081**	**59952139**	**6.77**	**1.29E-11**
**GTEx7_Whole_Blood**	***MS4A6A***	**11**	**59939081**	**59952139**	**6.55**	**5.93E-11**
GTEx7_Whole_Blood	*MYBPC3*	11	47352957	47374253	3.29	9.97E-04
YFS.BLOOD	*PLIN2*	9	19108373	19149288	-1.51	1.30E-01
NTR.BLOOD	*PLIN2*	9	19108373	19149288	-2.36	1.82E-02
**YFS.BLOOD**	***PTK2B***	**8**	**27168999**	**27316903**	**5.05**	**4.33E-07**
**NTR.BLOOD**	***PTK2B***	**8**	**27168999**	**27316903**	**5.90**	**3.74E-09**
**GTEx7_Whole_Blood**	***PTK2B***	**8**	**27168999**	**27316903**	**4.95**	**7.30E-07**
**GTEx7_Whole_Blood**	***PVR***	**19**	**45147098**	**45166850**	**-3.93**	**8.54E-05**
CMC.BRAIN	*PVR*	19	45147098	45166850	-5.98	2.19E-09
YFS.BLOOD	*SPI1*	11	47376411	47400127	3.88	1.06E-04
YFS.BLOOD	*STX3*	11	59480929	59573354	-0.80	4.23E-01

Supplementary Table 7. Transcriptome-wide association study (TWAS) test statistics for genes in blood and brain.

Test statistics are shown for genes in NTR blood, YFS blood, GTEx Whole blood and CMC brain (DLPFC) that were TWAS-significant in monocytes. K_TWAS.Z denotes the gene-level TWAS Z-score and K_TWAS.P denotes the TWAS p-value using the Kunkle 2019 summary statistics [5]. TWAS significant genes after multiple testing correction (Bonferroni) are shown in bold.

A. Logistic regression of AD disease status on genetically predicted PVR expression		
Variable	Beta	P
PVR expression	-3.74	0.0164
**B. Logistic regression of AD disease status on genetically predicted PVR expression conditional on number of APOE e4 alleles**		
Variable	Beta	P
PVR expression	-0.191	0.907
APOE e4 alleles	1.11	3.55×10^{-180}

Supplementary Table 8. PVR prediction is dependent on APOE genotype.

Allelic scores for the *PVR* gene were derived using the *PVR* expression weight from LPS24 monocytes and genetic data from the GERAD study. The following logistic regression analyses were performed: (A) AD disease status on genetically predicted *PVR* expression, (B) AD disease status on genetically predicted *PVR* expression correcting for the number of *APOE* e4 alleles. There is a significant negative association between the number of e4 alleles and *PVR* expression in (A). After correcting for the number of e4 alleles (B), the association of AD status with *PVR* expression is not significant. Therefore, the association of genetically predicted *PVR* expression with AD risk is explained by correlation with *APOE*.

Conditional analysis plots

Manhattan plots of the Kunkle 2019 GWAS data before (grey) and after (blue) conditioning on the green genes. SNPS are represented by coloured points plotted on the x axis by genomic location. The genes in the locus are represented at the top of the plot. Blue genes are marginally TWAS associated genes and green genes are jointly significant genes. The monocyte in which genes in the locus were TWAS-significant are shown in each plot.

1. PTK2B locus

Monocyte CD14, LPS24:

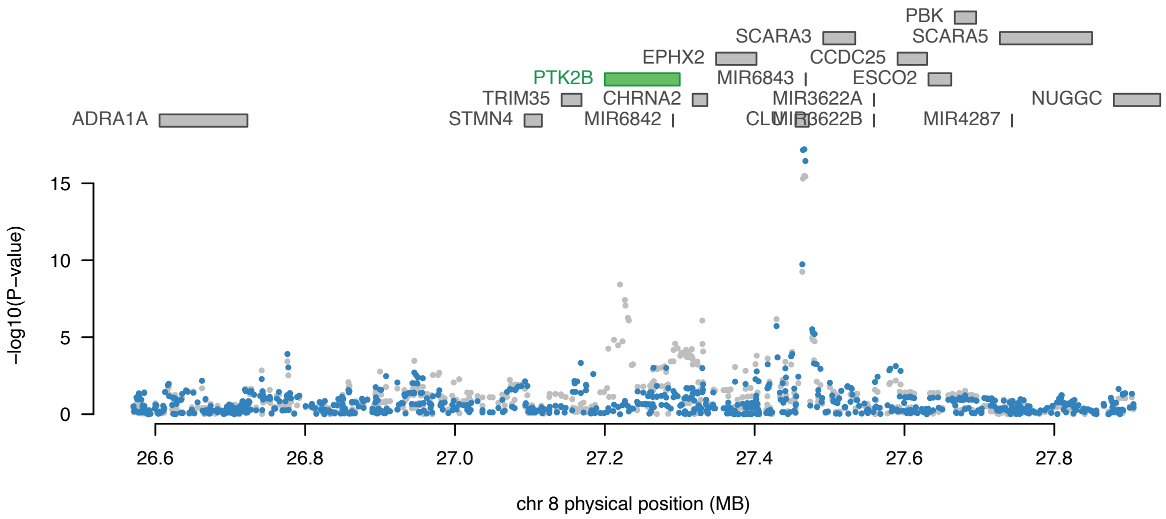

2. LACTB2 locus

Monocyte: CD14, IFN, LPS24.

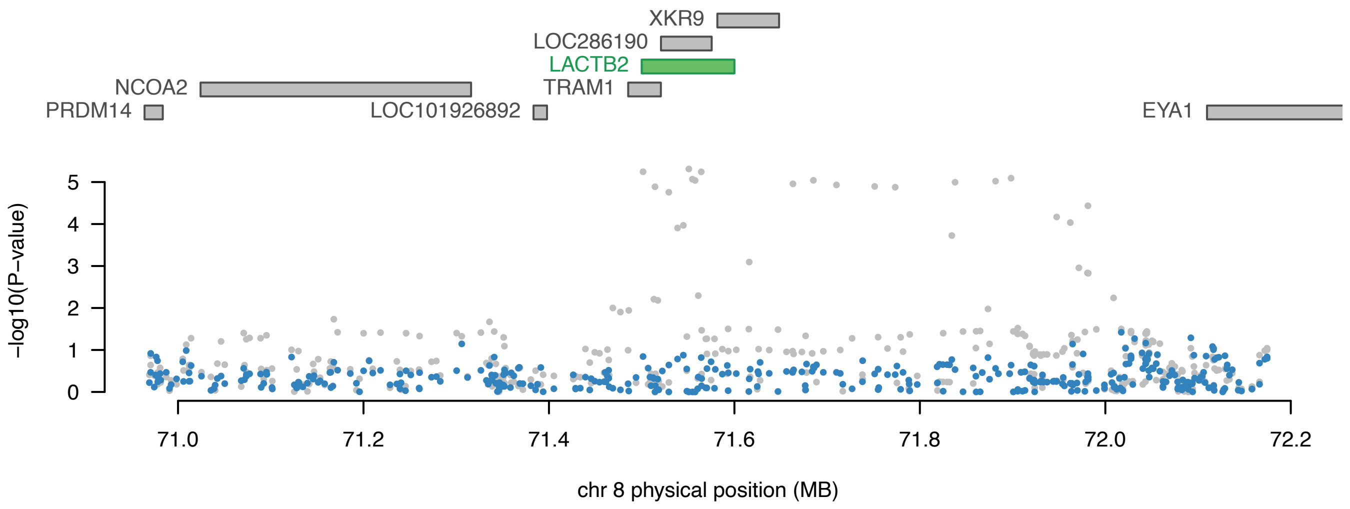

3. SPI1 locus

Monocyte: CD14, IFN, LPS2, LPS24.

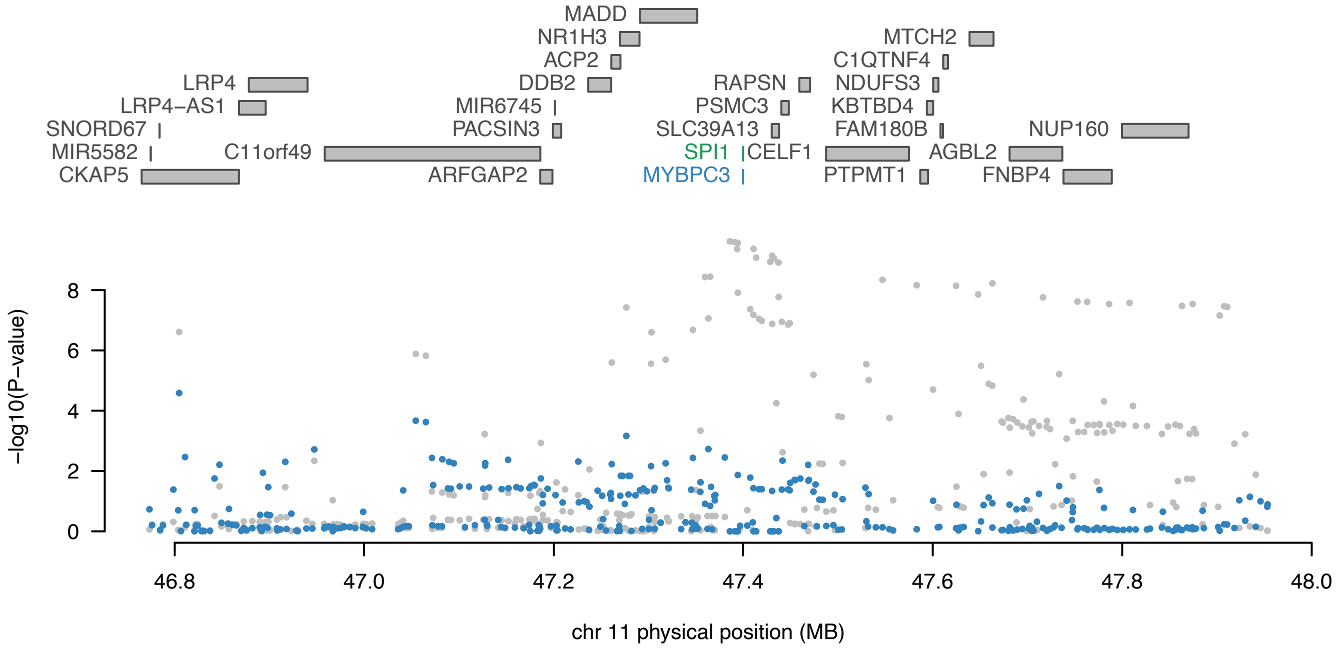

4A. MS4A4 locus

Monocyte: Fairfax CD14.

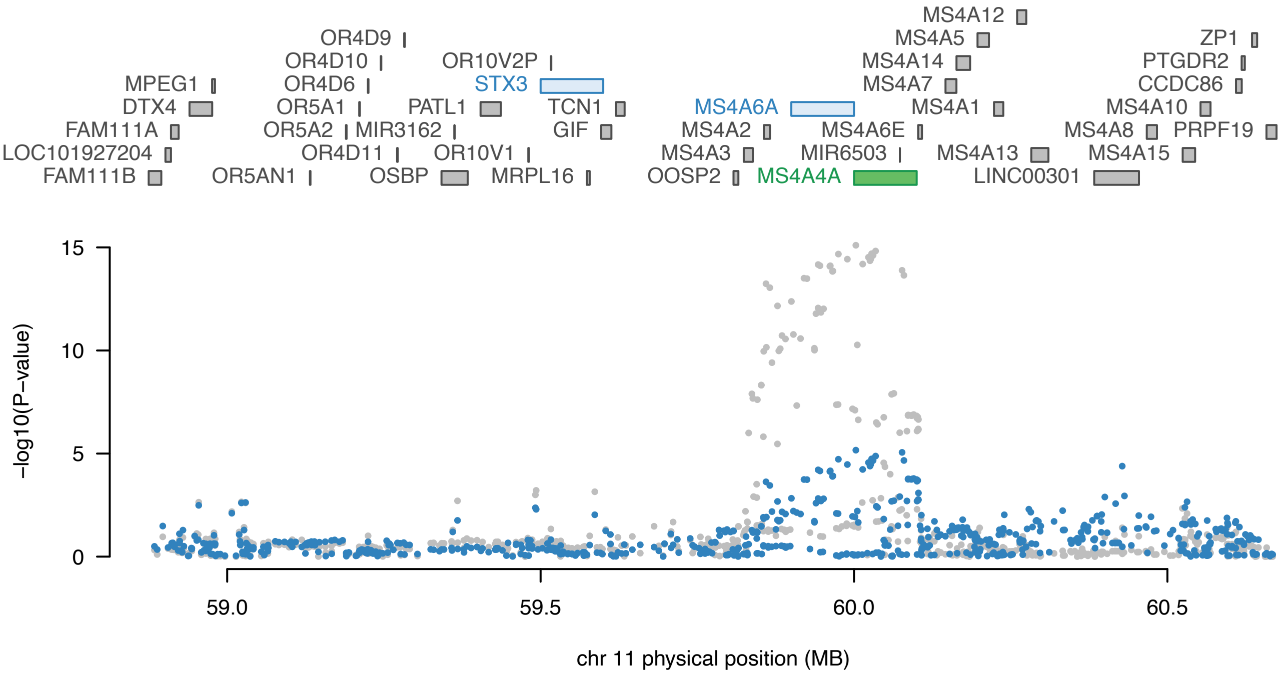

4B. MS4A4 locus

Monocyte: CTS CD14.

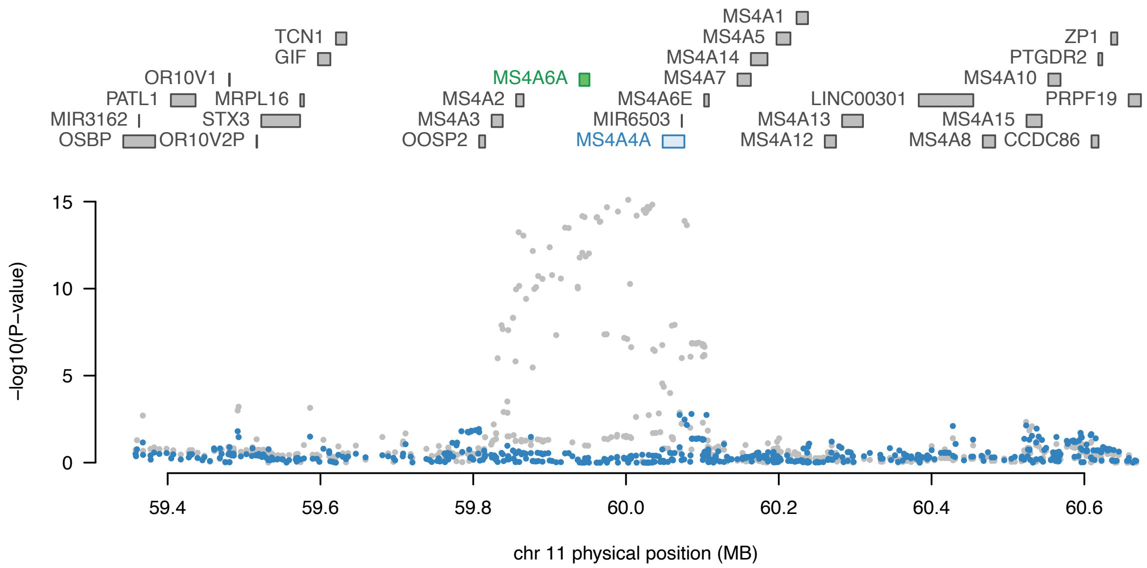

5. BIN1 locus

Monocyte: LPS2, LPS24

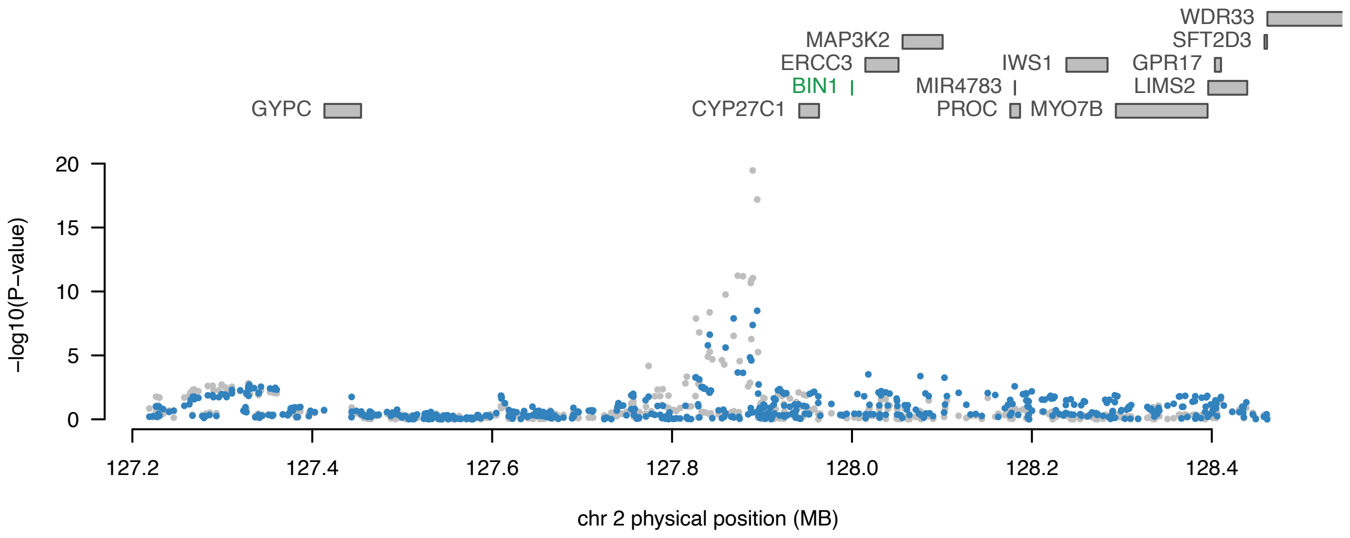

6. PLIN1 locus

Monocyte: LPS2.

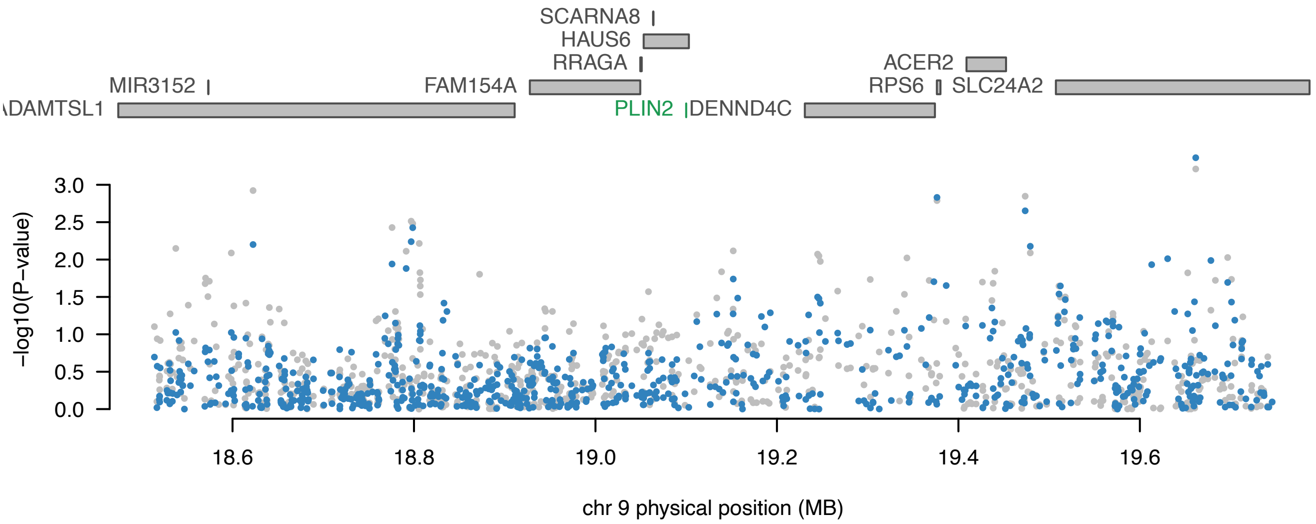

7. MS4A6E locus

Monocyte: LPS2, LPS24

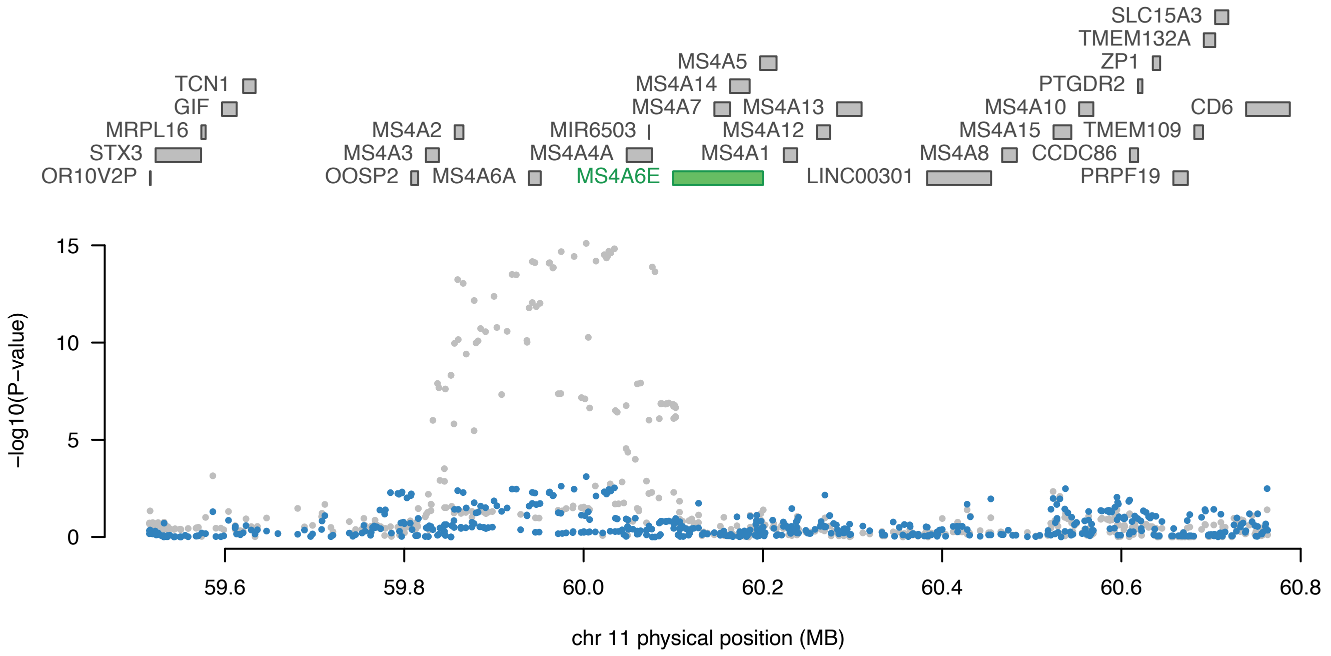

8. APOE locus

Monocyte: LPS2

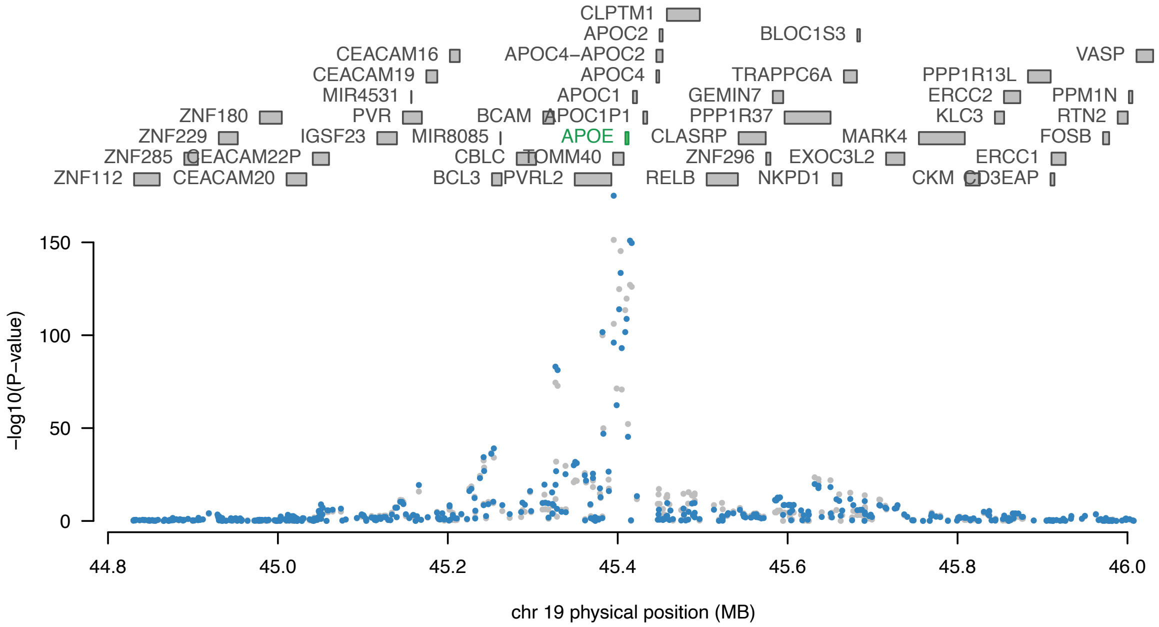

9. PVR locus

Monocyte: LPS24

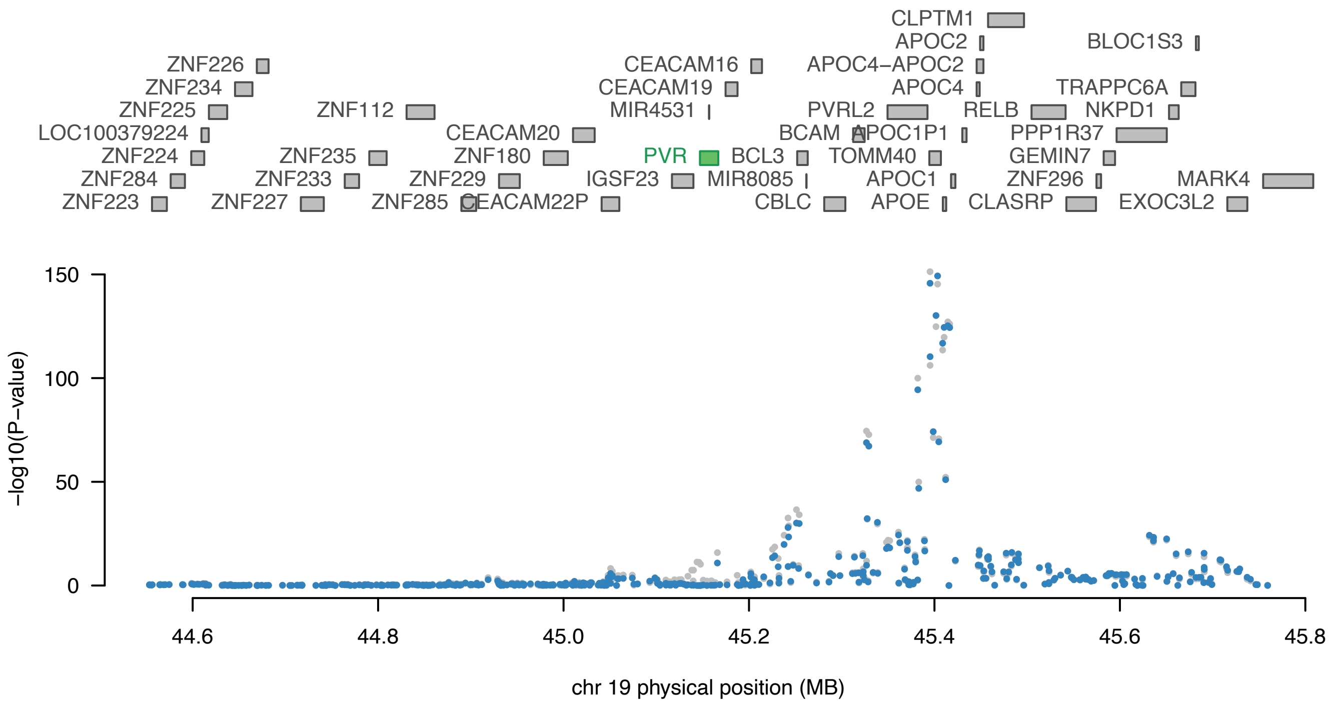

Supplementary references.

1. Farrer LA, Cupples LA, Haines JL, et al. Effects of age, sex, and ethnicity on the association between apolipoprotein E genotype and Alzheimer disease: A meta-analysis. *J Am Med Assoc*. 1997;278(16):1349-1356. doi:10.1001/jama.278.16.1349
2. Corder E, Saunders A, Strittmatter W, et al. Gene dose of apolipoprotein E type 4 allele and the risk of Alzheimer's disease in late onset families. *Science (80-)*. 1993;261(5123):921-923. doi:10.1126/science.8346443
3. Lv H, Zhang M, Shang Z, et al. Genome-wide haplotype association study identify the FGFR2 gene as a risk gene for Acute Myeloid Leukemia. *Oncotarget*. 2017;8(5):7891-7899. doi:10.18632/oncotarget.13631
4. Fairfax BP, Makino S, Radhakrishnan J, et al. Genetics of gene expression in primary immune cells identifies cell type-specific master regulators and roles of HLA alleles. *Nat Genet*. 2012;44(5):502-510. doi:10.1038/ng.2205
5. Kunkle BW, Grenier-Boley B, Sims R, et al. Genetic meta-analysis of diagnosed Alzheimer's disease identifies new risk loci and implicates Aβ, tau, immunity and lipid processing. *Nat Genet 2019 513*. 2019;51(3):414. doi:10.1038/s41588-019-0358-2
6. Huang KL, Marcora E, Pimenova AA, et al. A common haplotype lowers PU.1 expression in myeloid cells and delays onset of Alzheimer's disease. *Nat Neurosci*. 2017;20(8):1052-1061. doi:10.1038/nn.4587
7. Scott EW, Simon MC, Anastasi J, Singh H. Requirement of transcription factor PU.1 in the development of multiple hematopoietic lineages. *Science (80-)*. 1994;265(5178):1573-1577. doi:10.1126/science.8079170
8. Marioni RE, Visscher PM, Harris SE, et al. GWAS on family history of Alzheimer's disease. *Transl Psychiatry*. 2018;8(1):99. doi:10.1038/s41398-018-0150-6
9. Fairfax BP, Humburg P, Makino S, et al. Innate Immune Activity Conditions the Effect of Regulatory Variants upon Monocyte Gene Expression. *Science (80-)*. 2014;343(6175):1246949-1246949. doi:10.1126/science.1246949
10. Garnier S, Truong V, Brocheton J, et al. Genome-Wide Haplotype Analysis of Cis Expression Quantitative Trait Loci in Monocytes. Gibson G, ed. *PLoS Genet*. 2013;9(1):e1003240. doi:10.1371/journal.pgen.1003240
11. Seshadri S, Fitzpatrick AL, Ikram MA, et al. Genome-wide analysis of genetic loci associated with Alzheimer disease. *JAMA - J Am Med Assoc*. 2010;303(18):1832-1840. doi:10.1001/jama.2010.574
12. Lambert JC, Ibrahim-Verbaas CA, Harold D, et al. Meta-analysis of 74,046 individuals identifies 11 new susceptibility loci for Alzheimer's disease. *Nat Genet*. 2013;45(12):1452-1458. doi:10.1038/ng.2802
13. Karch CM, Ezerskiy LA, Bertelsen S, et al. Alzheimer's disease risk polymorphisms regulate gene expression in the ZCWPW1 and the CELF1 loci. Huang Q, ed. *PLoS One*. 2016;11(2):e0148717. doi:10.1371/journal.pone.0148717
14. Katsumata Y, Nelson PT, Estus S, Fardo DW. Translating Alzheimer's disease–associated polymorphisms into functional candidates: a survey of IGAP genes and SNPs. *Neurobiol Aging*. 2019;74:135-146. doi:10.1016/J.NEUROBIOLAGING.2018.10.017
15. Naj AC, Jun G, Beecham GW, et al. Common variants at MS4A4/MS4A6E, CD2AP, CD33 and EPHA1 are associated with late-onset Alzheimer's disease. *Nat Genet*. 2011;43(5):436-443. doi:10.1038/ng.801
16. Hollingworth P, Harold D, Sims R, et al. Common variants at ABCA7, MS4A6A/MS4A4E, EPHA1, CD33 and CD2AP are associated with Alzheimer's

disease. *Nat Genet*. 2011;43(5):429-436. doi:10.1038/ng.803
17. Jansen IE, Savage JE, Watanabe K, et al. Genome-wide meta-analysis identifies new loci and functional pathways influencing Alzheimer's disease risk. *Nature Genetics*. http://www.nature.com/articles/s41588-018-0311-9. Published March 7, 2019. Accessed March 20, 2019.
